# Effects of Metal Promoters (M = Fe, Co, and Cu) in Pt/M_*x*_Zr_*y*_O_*z*_ Catalysts and Influence of CO_2_ and H_2_O on the CO Oxidation Activity (PROX): Analysis of Surface Properties After Reaction

**DOI:** 10.1021/acsomega.3c09039

**Published:** 2024-06-07

**Authors:** Carolina C. Gaioto, José Carlos Pinto, Martin Schmal

**Affiliations:** † Programa de Engenharia Química/COPPE, Universidade Federal do Rio de Janeiro, Cidade Universitária, CP: 68502, Rio de Janeiro 21941-972 RJ Brazil

## Abstract

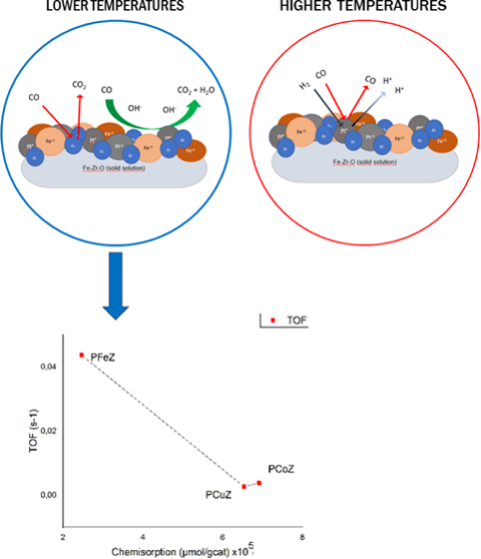

In the present paper, the effects of metal promoters (M = Fe, Co, and Cu) in Pt/M_*x*_Zr_*y*_O_*z*_ catalysts and the influence of CO_2_ and H_2_O on the CO oxidation activity (PROX) were investigated. To do that, characterizations of catalyst structures and surfaces were performed and reported here. The catalyst Pt/Fe_*x*_Zr_*y*_O_*z*_ (PFeZ) was the most active at low temperatures among the analyzed ones. The addition of platinum caused strong interaction with the mixed oxide, affecting the structure and the surface composition, blocking basic sites, and thus preventing catalyst deactivation. Particularly, diffuse reflectance infrared Fourier transform spectroscopy (DRIFTS) results evidenced the formation of carboxylate and carbonate species. Besides, the addition of CO_2_ and H_2_O in the gas feed stream affected the observed CO oxidation results, showing that CO_2_ competes with O_2_ on metallic sites. Moreover, DRIFTS and temperature-programmed desorption (TPD) analyses suggested the occurrence of OH^–^ oxidation by CO, leading to the formation of highly reactive compounds that can be easily oxidized.

## 1 Introduction

Planet Earth is facing dramatic climate changes due to the widespread use of energy produced from nonrenewable sources, making it possible to say that fossil fuels are the main cause of environmental imbalance. With the increasing energy demand and forecasts of future energy and climate crises, the search for new alternatives has become indispensable for sustainable development.^1^ For these reasons, many countries are implementing policies to reduce greenhouse gas emissions and investing in clean energy technologies. In this context, hydrogen production from renewable sources can constitute a competitive alternative and has attracted rising academic and commercial interests. For illustrative purposes, the production of hydrogen in 2019 was equal to 0.35 Mt/year and is estimated to reach 7.92 Mt/year in 2030.^2,3^

In particular, producing clean energy from syngas and hydrogen feedstocks in fuel cells can simultaneously enhance the efficiency of energy production, supply clean fuels, and mitigate the emission of pollutants to the atmosphere.^4,5^ However, residual contaminants from the autothermal reforming reactions (such as CO, CO_2_, and H_2_O), depending on the analyzed concentrations and operation conditions, can drastically reduce the performances of fuel cells.^6,7^ Therefore, several methods have been investigated to remove these contaminants from fuel cell streams, such as adsorption including powder-activated carbon, (PAC), and pressure swing adsorption, (PSA), membrane separation, scrubbing, and preferential oxidation (PROX).

Modern fuel cell membranes tolerate a maximum concentration of 50 ppm of CO in the feed stream. Therefore, the rich hydrogen gas mixture must be purified. To do that, the selective oxidation of CO should be performed, which can be regarded as the most appropriate method for small-scale production and mobile applications.^8^ One major competitive advantage of PROX is the possibility of converting a modest amount of CO in the presence of high H_2_ concentrations. In addition, this reaction can be carried out at low temperatures and atmospheric pressure.^9,10^

Previous studies proposed the use of catalysts based on mixed oxides to perform the PROX reaction. The existence of vacancies in the catalyst structures allows oxygen atoms from the network to participate in the oxidation reaction.^11−16^ More specifically, the performance of iron-zirconia solid solutions has been investigated in these systems^17−21^ although the use of CoFe_2_O_4_ mixed oxide resulted in high CO conversions (about 80%) and good stability (during 30 h) at 250 °C in the CO-PROX reaction.^22^

The use of dual metal Fe–Co single sites on N-doped carbon supports has also been investigated by Wang,^17^ using various characterization techniques and density functional theory (DFT) calculations to explain the observed synergetic effects of Fe–Co sites on the PROX reaction. The authors concluded that CO adsorption occurs on the Co sites and that oxygen dissociation takes place on the Fe sites.

On the other hand, Pt_0.5_Co_0.5_ catalysts also showed good performances for CO oxidation at 80 °C, while DFT calculations evidenced that the CO oxidation was associated with electronic effects.^18^ Besides, it has also been concluded that the strong synergetic effect between Pt and CoO_*x*_ enhanced the catalyst activity when compared to the Pt-FeO_*x*_ catalyst.^19^ Moreover, when Co was incorporated into CuO–Ce_0.8_Zr_0.2_O_2_ mixed oxide, the reaction activity could be increased even at lower temperatures, probably because Cu oxide species were well dispersed in the matrix, generating active sites.^20^ However, the addition of Co also favored the occurrence of undesired parallel reactions.^21^

More recently, computational and experimental studies have been performed to better comprehend the reaction mechanisms, including the influence of the reducibility of Cu on the reaction rates and the effects of lattice modifications on the structure of the support, which can affect the role of oxygen mobility through vacancies.^23^ For example, Dasireddy et al.^24^ studied the influence of Fe loading in Cu–Fe phases and its effect on carbon monoxide (CO) oxidation in H_2_-rich reactant streams. The Fe and Cu were found to be incorporated into a Cu–Fe supersaturated solid solution, which improved the CO oxidation activity, with carbon dioxide and water produced selectively with high catalytic activity in depleted hydrogen streams. The preferential oxidation of CO over CoFe_2_O_4_ promoted with M/CoFe_2_O_4_ (M = Ce, Co, Cu, or Zr) was studied by Béjaoui et al.^22^

Indeed, the mixed oxides have structures and morphologies that are active in the PROX reaction and other oxidation processes. The vacancies within mixed oxide compounds favor the oxidation process, depending upon the specific metal oxides or materials utilized to increase oxygen storage within the lattice framework of the oxide. However, the influence and effect of these promoters on the oxidation reactions have not been evaluated thoroughly, particularly when the feeds also contained CO_2_ and water.

Based on the previous paragraphs, in the present work, the influence of different metal oxide promoters added to mixed oxides on the performance of the PROX reaction is investigated. Reactions are carried out at relatively low temperatures, and the effects of impurities and undesirable products are analyzed. In order to do that, mixed oxides M_*x*_Zr_*y*_O_*z*_ were synthesized with promoters (M = Fe, Co, and Cu) by coprecipitation and impregnated with Pt as active sites by wetness point impregnation. These materials were then characterized by several techniques and tested in the preferential oxidation of CO and *in situ* diffuse reflection infrared Fourier transform (DRIFT) spectroscopy, in order to unveil the surface mechanism. The influence of contaminants on the conversions and selectivity for the Pt/Fe_0.25_Zr_0.75_O_*x*_ catalyst was also investigated by adding 10 vol % CO_2_ and 3 vol % H_2_O in the feed mixture. Finally, analysis of spent catalysts was performed to provide clues on the surface and structural modifications after the reaction.

## 2 Experimental Section

### 2.1 Materials

Zirconium(IV) oxynitrate hexahydrate ZrO(NO_3_)_2_·6H_2_O (purity of 99 wt %); iron(III) nitrate hydrate Fe(NO_3_)_3_·9H_2_O (purity of 99 wt %); hexachloroplatinic acid H_2_PtCl_6_·6H_2_O (purity of 99 wt %); and sodium hydroxide NaOH (purity of 98 wt %) were purchased from Sigma-Aldrich (São Paulo, Brazil). All chemicals were used as received without any further purification. Distilled water was used for all of the preparations.

### 2.2 Catalyst Preparation

Me_*x*_Zr_*y*_O_*z*_ mixed oxides were prepared by the coprecipitation method, starting from ZrO(NO_3_)_2_·6H_2_O, Co(NO_3_)_2_·6H_2_O, Cu(NO_3_)_2_·6H_2_O, and Fe(NO_3_)·9H_2_O as precursors.^12,15,25^ The appropriate amounts of precursor nitrates were initially dissolved in distilled water with a molar ratio of Me/Zr = 1:3. The resulting solutions were then thoroughly mixed with a magnetic stirrer to achieve homogeneous conditions; afterward, a basic aqueous solution containing NH_4_OH (25 wt %) was gradually added to the mixtures to increase the pH to 10.4. The resulting suspensions were filtrated and washed several times with distilled water until reaching the filtrate pH of 7.0. Subsequently, the filtrated precipitates were dried in a muffle at 90 °C overnight and finally calcined at 500 °C (heating rate of 2 °C/min) for 2 h under a static atmosphere. The obtained dry solid samples were finally named as M_*x*_Zr_*y*_O_*z*_.

Platinum (1.5 wt %) was impregnated by incipient wetness onto the prepared mixed oxides, using H_2_PtCl_6_·6H_2_O as a metal salt precursor. After impregnation, the samples were dried in a muffle furnace at 90 °C overnight, followed by calcination at 500 °C for 2 h and under continuous airflow (50 mL/min) to decompose the precursor salts. The resulting catalysts were named Pt/M_*x*_Zr_*y*_O_*z*_.

### 2.3 Catalyst Characterization

The chemical compositions were determined by X-ray fluorescence (XRF) spectroscopy using a Rigaku RIX 3100 model apparatus (Rigaku, Tokyo, Japan) equipped with a rhodium standard tube as a source of radiation.

The textural properties were measured by nitrogen physisorption experiments at liquid nitrogen temperature (−196 °C) using a Micromeritics ASAP 2020 instrument (Micromeritics, Norcross). Prior to the measurements, all samples were degassed at 300 °C overnight to remove the adsorbed species. The specific surface area was obtained using the well-known Brunauer–Emmett–Teller (BET) method^26−28^ in a relative pressure range of 0.05–0.3, whereas the pore size distributions were calculated by the Barrett–Joyner–Halenda (BJH) method.^29,30^

CO chemisorption experiments were carried out in a Micromeritics ASAP2020 automated gas sorption analyzer (Micromeritics, Norcross). Prior to the measurements, the samples were degassed at 250 °C for 30 min, followed by reduction under a H_2_ atmosphere at 500 °C for 30 min. Then, the samples were evacuated under vacuum for 30 min at the specified reduction temperature to remove any residual H_2_, before cooling to room temperature under vacuum for analysis.

To identify the crystalline phases, X-ray diffraction (XRD) patterns were recorded in a Rigaku Miniflex diffractometer (Rigaku, Tokyo, Japan), coupled to the data acquisition system. The experiments were performed in a 2θ range of 10–70° with a step size of 0.05°/min using Cu-Kα radiation (λ = 1.5406 Å). The operation beam voltage and current were set to 30 kV and 15 mA, respectively. Crystalline phases were identified with the help of the Joint Committee on Powder Diffraction Standards (JCPDS) database.

Surface analyses were performed by X-ray Photoelectron Spectroscopy (XPS) using an ESCALAB 250 spectrometer (Thermo Scientific, Waltham) equipped with Al Kα (1486.6 eV) as an X-ray source. The C 1s signal at 284.6 eV from adventitious carbon was used as a reference for the binding energy scale. Spectra were analyzed through the deconvolution of Gaussian–Lorentzian peaks with the help of CasaXps software.^31^

The morphology and microstructure of the catalyst samples were examined by scanning electron microscopy (SEM) using FEG-SEM QUANTA 400 equipment (FEI Company, Hillsboro). Elemental mappings were obtained with energy-dispersive X-ray spectroscopy (EDS) coupled to SEM.

Hydrogen temperature-programmed reduction (H_2_-TPR) analyses were carried out using H_2_/Ar 1.53/98.47 vol % mixture at a flow rate of 30 mL/min. The temperature was increased from room temperature to 1000 °C with a heating rate of 10 °C/min. The rate of hydrogen consumption during reduction was estimated with the help of a thermal conductivity detector (TCD). Prior to TPR analyses, samples were dried at 150 °C during 1 h of underflow of argon.

Temperature-programmed desorption (TPD) analyses were performed in a multipurpose unit equipped with a quadrupole mass spectrometer (Balzers Primas-QMS 200, Pfeifer, Memmingen, Germany). CO and CO_2_ desorption analyses comprised five steps: (i) treatment (100 mg of sample; He flow at 30 mL/min; heating rate of 10 °C/min until 250 °C, keeping this temperature constant for an additional 30 min); (ii) reduction (flow of H_2_/Ar 10:90 vol % at 30 mL/min; heating rate of 10 °C/min until 500 °C, keeping this temperature constant for additional 30 min); (iii) cooling to room temperature and cleaning with He flow at 30 mL/min for 90 min; (iv) adsorption CO/CO_2_/He 1:1:98 vol % constant flow of 30 mL/min for 30 min at 25 °C and atmospheric pressure; (v) cleaning (He flow of 30 mL/min at 25 °C for 60 min); (vi) desorption (linear temperature increase from room temperature to 500 °C with a heating rate of 20 °C/min).

Temperature-programmed oxidation (TPO) analyses were carried out after the CO-TPD tests, heating from room temperature to 500 °C at 10 °C/min with a flow of 30 mL/min of 5:95 vol % of O_2_/He to identify the existence of carbon after the reaction.

Diffuse reflectance infrared Fourier transform spectroscopy (DRIFTS) analyses were performed in a closed chamber using a Nicolet Nexus 470 (Thermo Scientific, Waltham), equipped with an MCT-A ZnSe detector, with a resolution of 4 cm^–1^ and wavelength numbers ranging from 650 to 4000 cm^–1^. Analyses were also performed for flows of mixed gases with a Spectrum 100 Fourier transform infrared (FT-IR) Spectrometer (PerkinElmer, Waltham), equipped with an MCT-A ZnSe detector, with a resolution of 4 cm^–1^ and wavelength numbers ranging from 650 to 4000 cm^–1^. The analyses were carried out for mixtures of CO/He 1:99 vol %, CO/O_2_/He 1:1:98 vol %, and CO/O_2_/H_2_/He 1:1:60:38 vol % at 90, 100, and 120 °C for the Fe_0.25_Zr_0.75_O_*x*_ mixed oxide (FeZ) and at 90, 100, 120, 170, and 200 °C for the Pt/Fe_0.25_Zr_0.75_O_*x*_ catalyst (PFeZ).

Pyridine infrared analyses were performed for the determination of Brönsted and Lewis sites using a Nicolet Nexus 470 (Thermo Scientific, Waltham), equipped with an MCT-A ZnSe detector, with a resolution of 4 cm^–1^ and wavelength numbers ranging from 650 to 4000 cm^–1^.

Raman spectroscopy was realized before and after the reaction using the LabRam HR-UV800/Jobin-Yvon, 1 μm^3^, He–Ne laser λ = 632 nm, with a detector temperature at −70 °C and an Olympus BX41 microscope with 50× and 100× objective lenses.

### 2.4 Catalytic Tests

The catalytic tests were carried out in a fixed-bed U-shaped quartz reactor, coupled to a gas Varian CP3800 chromatograph (GC, Cole-Parmer, Vernon Hills) equipped with flame ionization detector (FID) and a thermal conductivity detector (TCD) connected in series and using a molecular sieve Poraplot-Q column under a He flow of 25 mL/min. Besides, all catalytic tests were performed at atmospheric pressure.

Before the tests, the catalysts were dried under a He flow (heating rate of 10 °C/min, from room temperature to 250 °C). After drying, the temperature was reduced to room temperature and then reduced under H_2_ flow and heated until 500 °C (heating rate of 10 °C/min). Then, samples were cleaned under a He flow for 30 min until room temperature.

The tests were performed using feed gas mixtures of CO/O_2_/H_2_/He (1:1:60:38 vol %) and CO/O_2_/CO_2_/H_2_/He (1:1:10:60:28 vol %). About 100 mg of the catalyst sample was placed in the reactor at a feed flow rate of 120 mL/min. Test temperatures were set at 70, 100, 150, 200, and 250 °C. The data acquisition was performed in triplicate for the characterization of experimental variability, and outlet gas compositions were analyzed by GC as described previously.

The influence of water was studied by a temperature-programmed reaction, coupled to MS under similar conditions of the tests described above, by adding water to the feed stream: CO/O_2_/H_2_/H_2_O/He (1:1:60:3:35 vol %) and switching alternatively to the initial feed mixture under isothermal condition. First, without water and increasing temperature at 10 °C/min until reaching the set temperature and then alternating feed input, with and without water under isothermal conditions. Data analyses were formulated for the fixed-bed reactor and low conversions according to eqs 1–4.
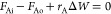
1where *F*_Ai_ and *F*_Ao_ are input and output molar flows of A, respectively, and
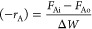
2where Δ*W* is the mass of the catalyst. As
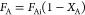
3then
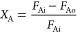
4where *X*_A_ is the conversion of A. Considering the high dilution of the feed and the low conversions, CO conversion (*X*_CO_), O_2_ conversion (*X*_O_2__), and CO_2_ selectivity (*S*_CO_2__) were calculated as follows:
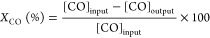
5
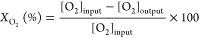
6
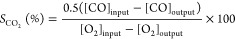
7

## 3 Results and Discussion

### 3.1 Chemical Composition and Textural Properties

The chemical composition was obtained by XRF measurements, as presented in Table S1. As seen, the experimentally measured Pt values were in good agreement with the nominal contents, and the observed differences were within the experimental error. This finding indicates the effectiveness of the preparation procedure. Conversely, the chemical composition of the Zr oxide sample was slightly different from the nominal values, which can be attributed to the loss of material during the filtration and washing steps. It must also be noted that no chlorine was observed in the samples.

The nitrogen adsorption/desorption isotherms are shown in the Supportin Information (Figure S1). In all cases, the obtained isotherms presented the standard H-type hysteresis shapes, according to the classification of IUPAC,^30^ indicating the mesoporous character of the materials.

The textural parameters obtained from the N_2_ physisorption experiments are summarized in Table 1. BET analyses showed that specific areas decreased (from 156 to 136 m^2^/g) and that the average pore diameters increased (from 48 to 53 Å) after platinum impregnation on the mixed oxide support. The CO chemisorption showed high CO adsorption on Pt sites.

**Table 1:** Specific Surface Areas, Pore Volumes (BET), and CO Chemisorption

sample	specific area (m^2^/g)	pore diameter (Å)	*S*_m_ (m^2^/g_metal_)	*D* (%)
FeZ	156	48	1.1	0.31
PFeZ	136	53	88	49.2
CoZ	95	67	1.4	0.4
PCoZ	70	77	240	50.1
CuZ	118	27	1.5	0.4
PCuZ	31	59	209.4	51.2

The specific areas of the supports and catalysts based on Fe, Co, and Cu are presented in Table 1. Details about the calculations of metallic surface areas and dispersions of the Pt catalysts are presented in Section S7 of the Supporting Information. One must note that the dispersions of all samples were of the order of 50%, while for the mixed oxides without Pt, these values were very low, about 0.4%, indicating a good and similar distribution of Pt on the different mixed oxides. Despite that, the specific surface areas of the mixed oxides are two orders of magnitude higher than those of the samples prepared with Pt, with the exception of the PFeZ sample. Indeed, these results are comparable to other values reported in the literature.^12,32−35^

### 3.2 X-ray Diffraction

**Figure 1:**
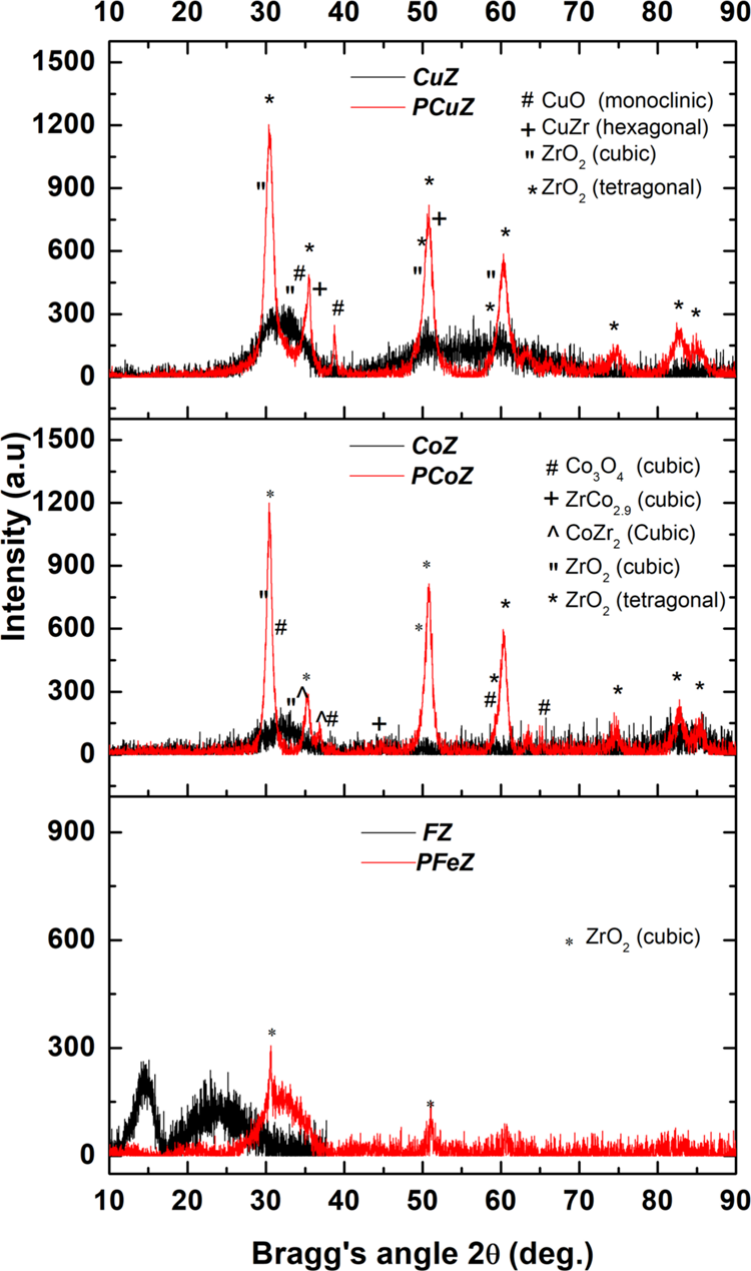
XRD diffractograms of the supports and catalysts, CuZ, CoZ, and FZ, and the corresponding catalysts, PCuZ, PCoZ, and PFeZ, respectively.

The diffraction patterns of the mixed oxides CuZ, CoZ, and FeZ and the corresponding catalysts PCuZ, PCoZ, and PFeZ, respectively, are displayed in Figure 1.

As one can see in Figure 1, the diffractograms of the mixed oxides (**black**) present characteristic amorphous phases. These results agree with the literature for similar brackets, containing 20–30 mol % of Fe,^12,15^ probably because of the formation of a solid solution. However, catalysts (**red)** prepared with Pt displayed characteristics of phase segregation. In the case of the PFeZ catalyst, ZrO_2_ segregation occurred in the cubic phase only in small amounts. For the PCoZ and PCuZ catalysts, ZrO_2_ segregations occurred in the cubic and monoclinic configurations, cubic phases of Co_3_O_4_ and CoZr_2_ for the PCoZ sample; monoclinic phase of CuO; hexagonal phase of CuZr on the PCuZ catalyst. The formation of these crystalline phases can also explain the reduction of the surface area after the addition of platinum, as also discussed in the literature.^12,15,25,31,32^

### 3.3 Temperature-Programmed Reduction

**Figure 2:**
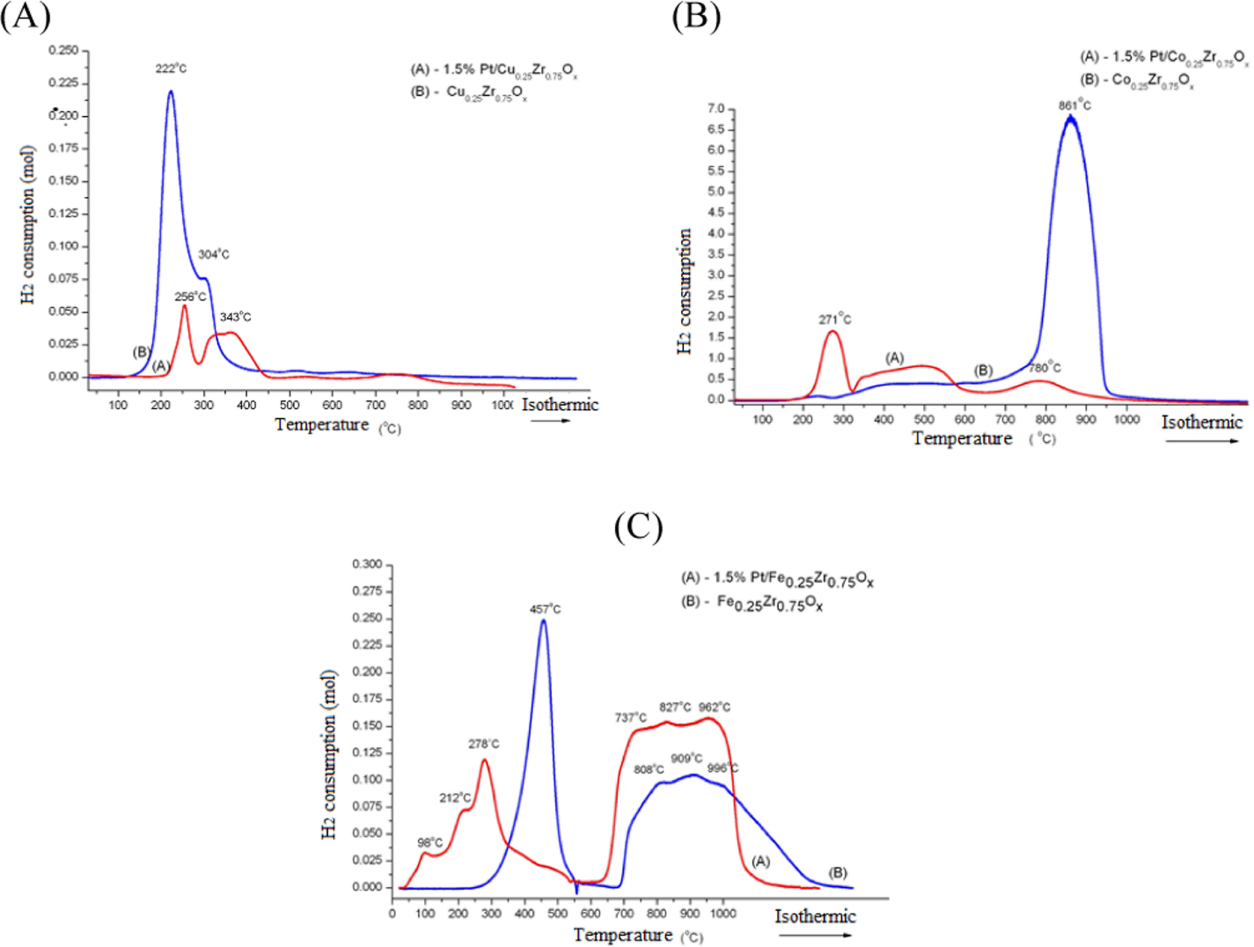
H_2_-TPR profiles for CuZ, PCuZ (A), CoZ, PCoZ (B), and FeZ, PFeZ (C) samples.

Figure 2 shows the hydrogen temperature-programmed reduction (H_2_-TPR) profiles of CuZ, PCuZ, CoZ, PCoZ, FeZ, and PFeZ materials.

The FeZ mixed oxide displayed a characteristic reduction of hematite to magnetite at 457 °C (Figure 2C)
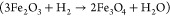
8

While the zirconium reduction occurred at higher temperatures,^12^ the Pt-supported catalyst (PFeZ) exhibited four reduction peaks between 20 and 600 °C: (i) the first one at 98 °C indicates the reduction of platinum oxide to metallic Pt^0^, (ii) the second peak at 212 °C is attributed to the interaction between Fe and Pt,^36^ (iii) the third peak at 278 °C can be assigned to the reduction of Fe^2+^ to Fe^0^ or the reduction of magnetite to metallic iron
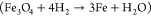
9and (iv) the fourth peak represents the reduction of Fe oxide (hematite) to magnetite
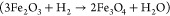
10

Besides, a peak that appeared at 737 °C can be related to the reduction of Zr^4+^ to Zr°, as reported in previous works.^12,37^

The cobalt oxide profile exhibited two characteristic reduction steps: (i) Co_3_O_4_ to CoO and (ii) to Co^0^. With Pt, part of ZrO_2_ was drawn from a mixed oxide matrix, as observed in the XRD pattern, and the unique peak disappeared. On the other hand, new peaks appeared at 271 °C, between 330 and 650 °C (relative to Co oxidation states) and 780 °C (related to Zr) (Figure 2B). It can be observed that metallic platinum facilitated the reduction of Co oxides.^5^ According to the XRD diffractogram, this can be assigned to the reduction of Co_3_O_4_ to CoO and the formation of CoZr_2_ phases. The CuZr mixed oxides exhibited peaks at 222 and 304 °C (Figure 2A), which indicate segregation of the ZrO_2_ phase, according to the literature.^5^Table S2 presents the hydrogen consumption of the FeZ and PFZ catalysts.

### 3.4 Catalytic Tests

**Figure 3:**
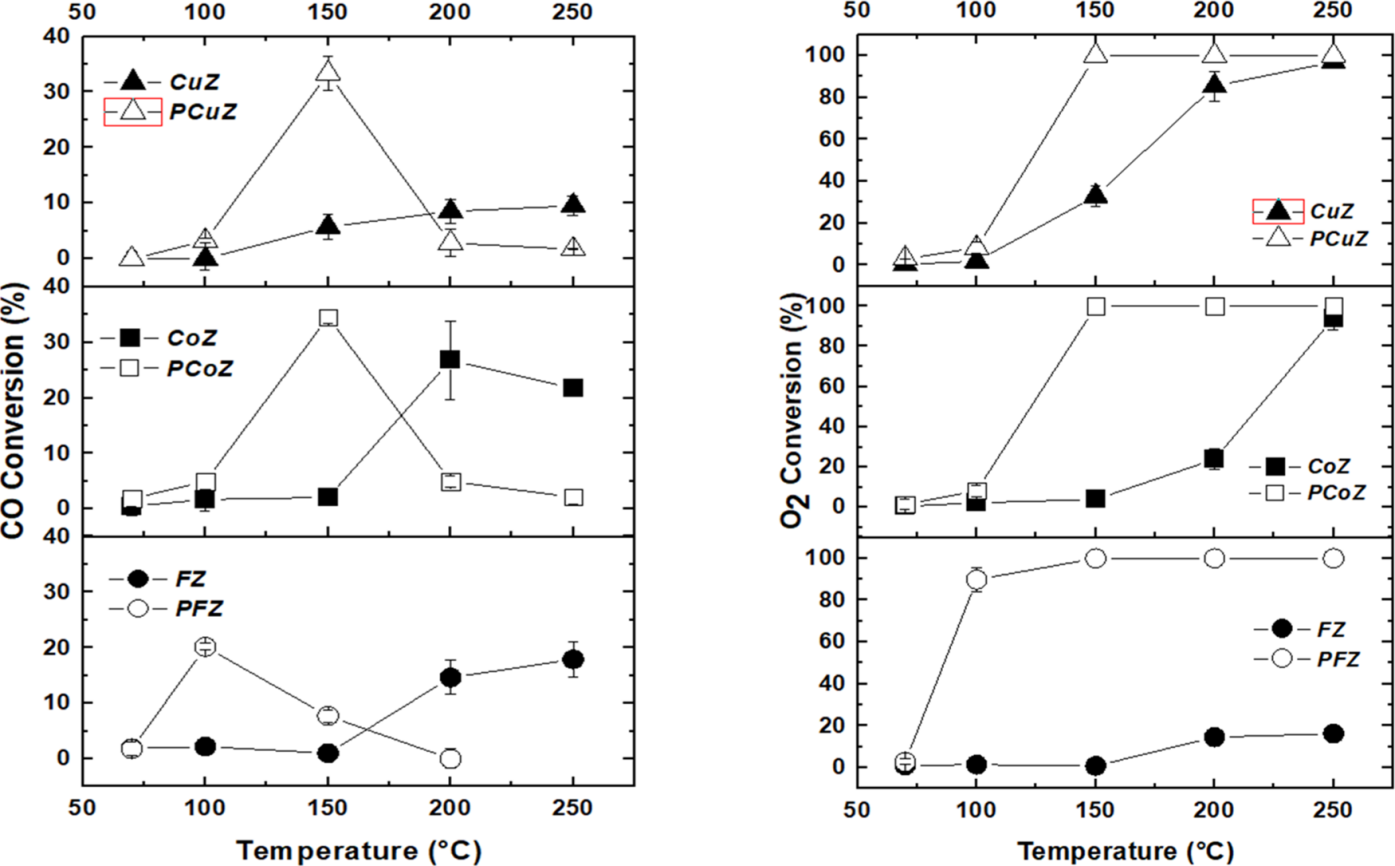
CO and O_2_ conversions for PROX reaction with feed composition of CO/O_2_/H_2_/He (1:1:60:28 mol %), *m*_cat_ = 100 mg, and total flow *F* = 120 mL/min.

The first catalytic tests were performed with a feed mixture (CO/O_2_/H_2_/He 1:1:60:38 vol %). Figure 3 displays the obtained CO and O_2_ conversions as functions of the reaction temperature. One must observe that the CO conversions for pure mixed oxides were very low (10%).

The maximum CO conversions for the analyzed reaction conditions were 20 and 30% at 100 and 150 °C, respectively. It is important to observe that the PFeZ catalyst provided maximum conversion at 100 °C, while catalysts PtCuZ and PtCoZ provided maximum conversions at 150 °C. The O_2_ conversions followed similar trends, reaching 90, and 100% at 100 and 150 °C, respectively. Meanwhile, the selectivity toward CO_2_ was reduced with the increasing temperature for all promoted catalysts due to the CO and H_2_ competition for active sites, which favors the oxidation of H_2_ (Figure S5).

The activity for all catalysts was calculated based on the reaction rates and experimental chemisorption results. The TOF values are presented in Table 2, while the calculation of the metallic surface area and dispersions are presented in the Supporting Information (S7).

**Table 2:** Catalyst Activities at 100 °C

catalyst	rate (mol/g_cat_·s) at 100 ^o^C	chemisorption (μmol/g_cat_) × 10^5^	TOF (s^–1^)
PFeZ	0.0107	2.466	0.0437
PCuZ	0.00176	6.534	0.00269
PCoZ	0.00269	6.911	0.00381

One must note that the PFeZ catalyst was the most active when compared to the PCoZ and PCuZ catalysts at 100 °C. Indeed, the TOF value of PFeZ was 10 times higher than that observed for the other catalysts, as seen in Table 2. As a matter of fact, at higher temperatures, other parallel reactions can occur, predominantly hydrogen oxidation and the Boudouard reaction, leading to the formation of carbon on the surface
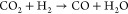
11
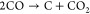
12

Regarding the activities of the analyzed mixed oxides, the PFeZ catalyst also provided the highest activity (TOF) and the maximum CO conversion at lower temperatures when compared to the other mixed oxides containing Co and Cu in its structure.

**Figure 4:**
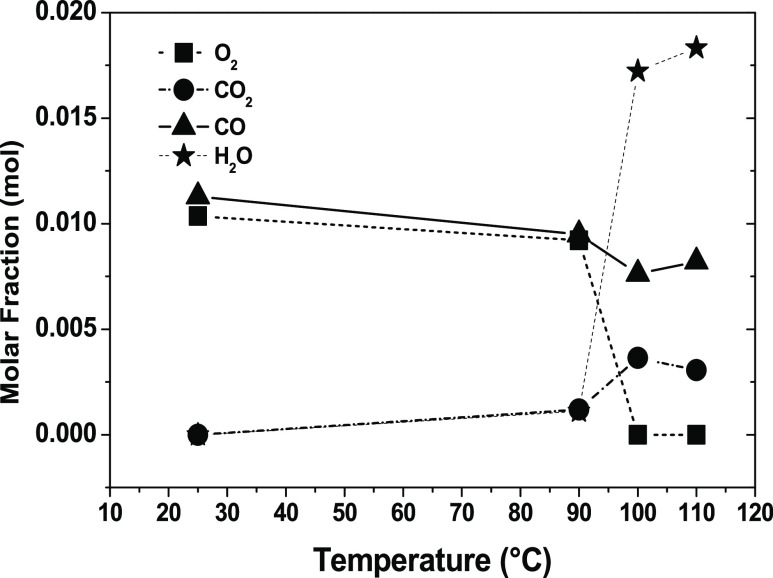
Outlet gas composition for the PFeZ catalyst with a feed flow rate of 200 mL/min.

Figure 4 displays the product distribution for the PFeZ catalyst with increasing temperature, with the expected formation of H_2_O and CO_2_. The marked difference could be noted above 90 °C and drastic change at 100 °C, with a sudden increase of water and CO_2_ compositions in the outlet stream.

### 3.5 Influence of CO_2_ and H_2_O on the Catalytic Activity

**Figure 5:**
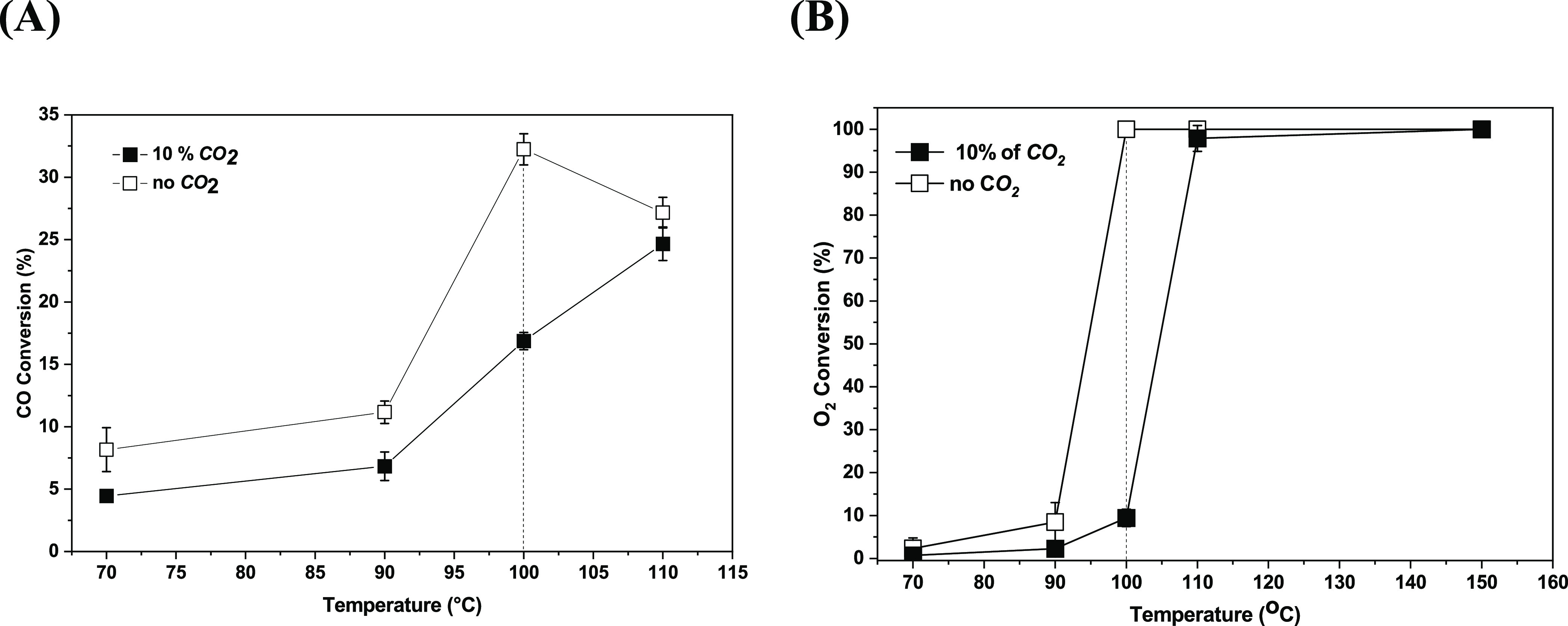
Influence of CO_2_ on (A) CO conversion and (B) O_2_ conversion (■) with and (□) without CO_2_ and using the PFeZ catalyst.

CO_2_ and H_2_O are products of the investigated reaction, but when present in the feed stream, they may influence the product distribution and activity. The influence of CO_2_ on the conversion was studied by adding CO_2_ to the feed stream. Tests were performed with the PFeZ catalyst with 10 vol % of CO_2_ in the feed composition (CO/O_2_/CO_2_/H_2_/He 1:1:10:60:28 vol %) at different temperatures. Figure 5A,5B display the CO and the O_2_ conversions, respectively, in the absence and presence of CO_2_ at increasing temperatures.

Obtained results showed marked differences on the CO and the O_2_ conversions. Between 70 and 90 °C the influence of CO_2_ was negative. This difference was mainly observed at 100 °C, when the CO conversion decreased from 32.2% to approximately 16.8% with and without CO_2_, and the O_2_ conversion decreased from 100 to 10%, respectively. This suggests preferential CO_2_ adsorption on the surface of the mixed oxide.

**Figure 6:**
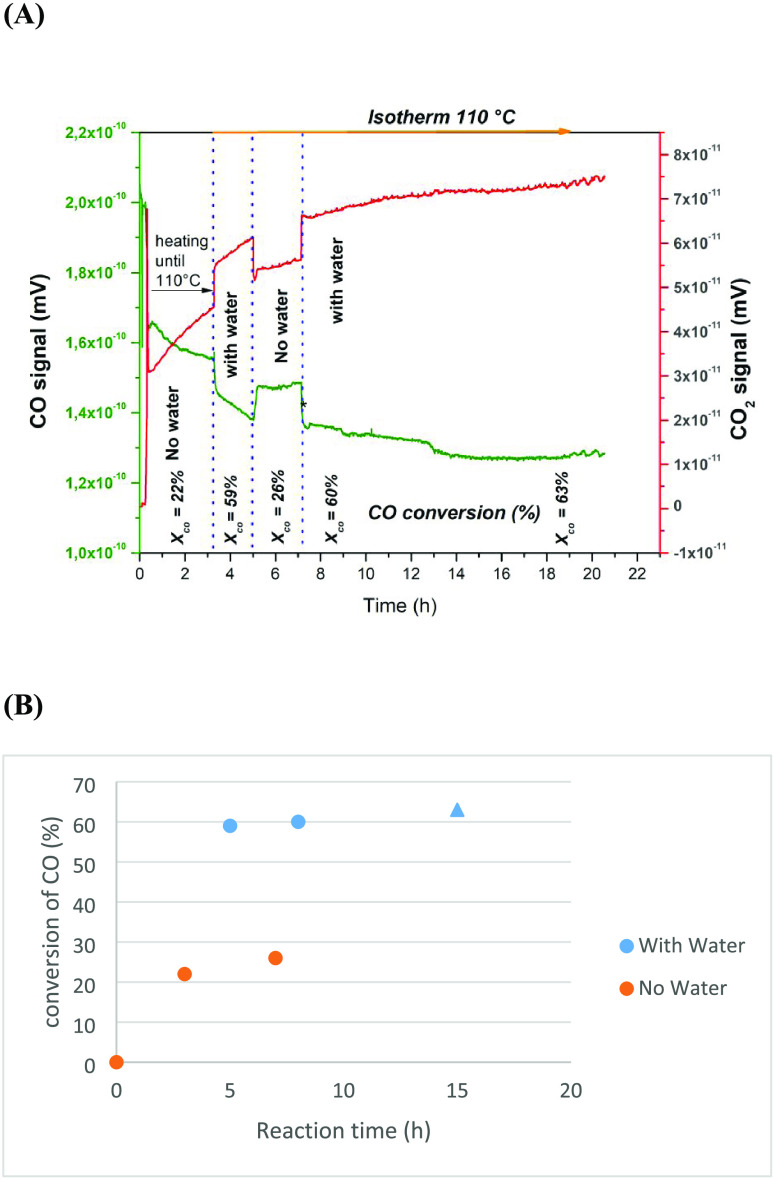
Influence of water on the conversion of CO as a function of time: (A) original time-on-stream signal and (B) conversion for the PFeZ catalyst.

The influence of water on the feed composition has been reported in the literature for this reaction, affecting both the catalyst activity and selectivity. The effect of water on the PFeZ catalyst was studied using the TPRe unit coupled to a mass spectrometer, as described in the previous section. The experiments were performed as described in the experimental part, by adding 3 vol % of H_2_O in the feed stream (CO/O_2_/H_2_O/H_2_ in He: 1:1:3:60:35 vol %). The compositions of all compounds were then monitored with TOS values, and conversions were determined during 23 h, as displayed in Figure 6. In the first stage, the temperature was increased to a maximum of 100 °C without water. Afterward, the feed stream was switched to the feed stream that contained water at a constant temperature for 20 h.

Figure 6A displays the CO conversion and the CO_2_ formation. In the first stage, with increasing temperature, the CO conversion reached 22% at 110 °C. When compared with the results observed in the fixed bed, without water (Section 3.4), the CO conversion was 20%, approximately the same value obtained in this first stage. Then, with water in the second stage and at a constant temperature, the CO conversion increased significantly, about 3 times, reaching 59% after 3 h. In the third stage, after removing water, the CO conversion decreased to 26% after approximately 8 h, very close to the first stage (20%). Finally, switching again to water in the feed stream, the CO conversion reached 63% and remained constant for 23 h. This conversion was very close to that observed in the second stage (59%). Figure 6B compares the CO conversions in the presence and absence of water in the feed stream, which suggests that water significantly influenced the activity. It is important to observe that the conversion of aqueous O_2_ at this temperature was very high, above 90% (not shown). The mixed oxide FZ sample was also analyzed under similar conditions, and results are shown in Figure S6 of the Supporting Information, displaying clearly a low influence of water on the conversion of CO with time on stream as a function of time.

Dasireddy et al.^24^ also showed the influence of CO_2_ and observed a significant effect on hydrogen oxidation and the consequent reduction of CO conversion, in good agreement with the results shown in Figure 5. In fact, the CO_2_ in the feed stream inhibits CO oxidation due to the formation of carbonates on the active sites of the metal oxide and the Boudouard reaction, which decomposes the CO into carbon over the surface and promotes deactivation. On the other hand, with the addition of water in the feed stream, the CO conversion increases (Figure 6), leading to CO_2_ formation, which can be attributed to the water gas shift reaction (WGSR). However, by removing water from the feed stream, the conversion decreased significantly, with the consequent reduction of the CO_2_ composition. In fact, the H_2_ oxidation prevailed at these conditions and the O_2_ conversion reached 100%, in accordance with the literature.^38^

### 3.6 Temperature-Programmed Desorption (TPD) Analyses

Figure S2(A,B) of the Supporting Information shows a broad CO_2_ peak located between 50 and 425 °C, with a maximum peak at 148 °C, and a CO profile with maximum desorption at 130 °C. In addition, above 200 °C, water desorption can also be observed. These results indicate that the dominant mechanism is CO oxidation, using oxygen from the lattice, leading to the formation of CO_2_ and vacancies. There are two underlying assumptions about the formation of water: (i) reactions among hydroxyl groups and (ii) reaction of CO molecules with hydroxyl groups to form carboxylates and carbonates, which can react with the oxygen lattice to produce CO_2_ and water.^14,15^ The CO-TPD experiments resulted in small amounts of C(s), probably associated with the Boudouard reaction, contributing to the formation of CO_2._^12^Table 3 presents the maximum temperature of desorption and the amounts of CO_2_ and CO desorbed of FZ and PFeZ catalyst samples.

**Table 3:** Global Results of TPD-CO Experiments

sample	peak temperature (°C)	CO_2_ desorption (μmol CO_2_ g_cat_^–1^)	CO desorption (μmol CO g_cat_^–1^)
FZ	148	111	1.98^a^
PFZ	112, 220	24	1.63^a^

a Calculated considering the removal of the contribution from the signal *m*/*e* = 28 from CO_2._

The CO_2_ desorption of the FZ support was equal to 111 μmol of CO_2_/g_cat_ and with the addition of Pt decreased significantly to 24 μmol of CO_2_/g_cat_. Simultaneously, the CO desorption rate was reduced from 1.98 to 1.63 μmol CO/gcat in the presence of Pt. Nevertheless, marked differences were observed for the support FZ and the catalyst PFeZ. In the first case, CO molecules seemed to adsorb, diffuse, and react with oxygen atoms in the lattice although CO molecules can also react with hydroxyl groups at the surface to form CO_2_, releasing higher amounts of CO_2_ when compared with the experiments performed with the Pt-supported catalyst. In fact, in this case, CO molecules can be partially adsorbed onto the metallic Pt^0^ particles formed on the surface. The release of CO from the FZ support can also be attributed to the formation of carbonates that are decomposed during the desorption process.

### 3.7 SEM and EDS Analyses

**Figure 7:**
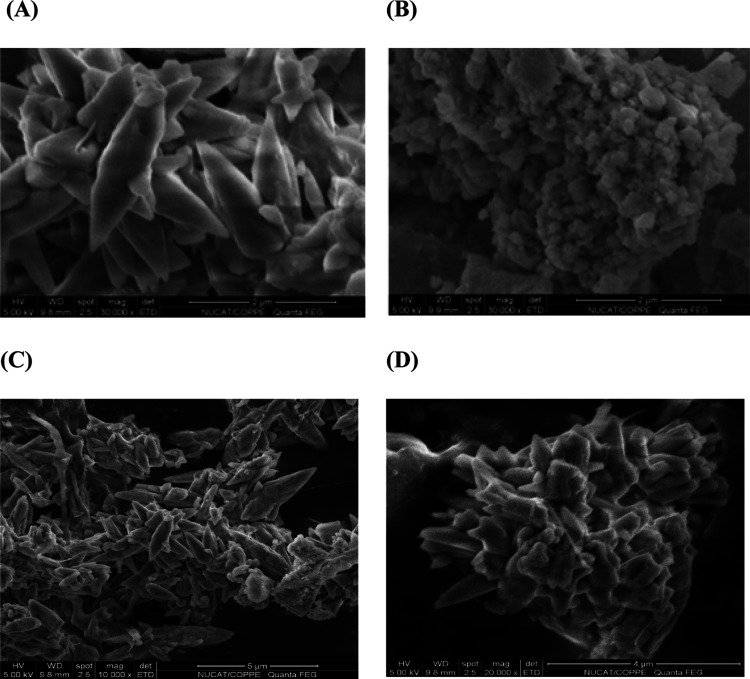
SEM micrographs of FeZ and PFeZ samples. Mixed oxides (A, C) and (B, D) structural changes of FeZ.

SEM images display the morphological structures of the oxides, as shown in Figure 7. The mixed oxides (Figure 7A,7C) present a triagonal structure, like nanorod crystallites. Figure 7B,D indicate structural changes of FeZ, leading to the formation of nanoflowers. The corresponding image of the catalyst (PFeZ) shows Pt particles well dispersed over the mixed oxide support and small crystallites with similar shapes.

Table 4 presents the elemental compositions, as determined by EDS analyses. The changes can be attributed to structural modifications during calcination. The nominal Pt content was equal to 1.5 wt % and is in good agreement with the experimental value of 1.3 wt %.

**Table 4:** Superficial Composition from EDS Analyses

	FeZ	PFeZ	FeZ	PFeZ
element	weight %		atomic %	
O	27.8	38.8	66.4	76.5
Fe	12.8	12.0	8.7	6.8
Zr	59.4	47.9	24.9	16.5
Pt		1.3		0.2
total	100.0	100.0	100.0	100.0

### 3.8 XPS Analyses

**Figure 8:**
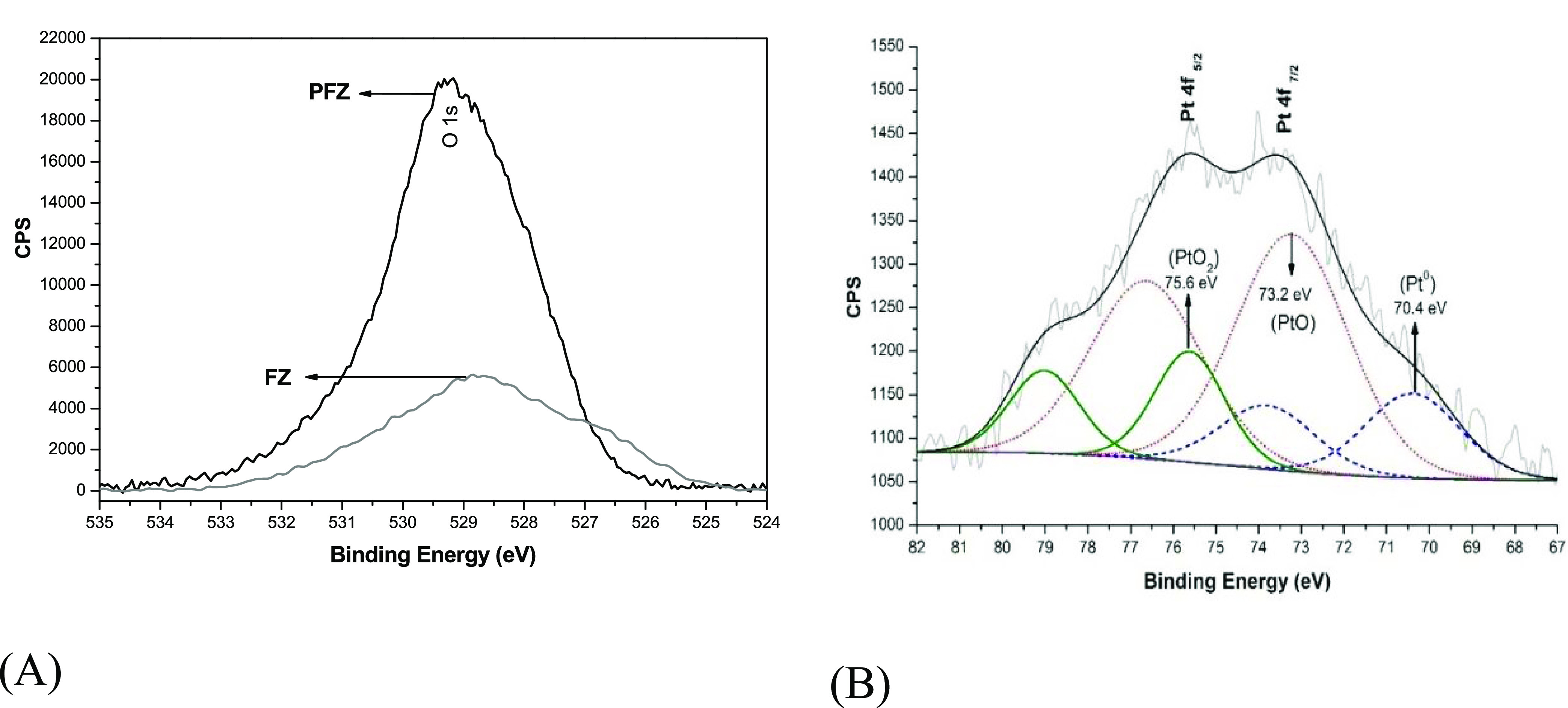
XPS of oxygen O 1s (A) and platinum (B) spectra of sample PFeZ.

XPS analyses were performed to examine the surface properties and the nature of the metallic oxide phases of the different catalysts. Particularly, the self-reorganization of Pt active sites depends on the synergetic interaction with electron donors from the support. The XPS analyses were performed using the C 1s peak as a reference. Figure 8A displays the oxygen O 1s spectrum of the FeZ support and the PFeZ catalyst. The FeZ support presents the band at 530 eV, which can be assigned to oxygen adsorption, and broad bands at lower binding energy levels that can be assigned to oxygen atoms present in the lattice of the mixed oxide.^39^ The XPS results also show the concentration of zirconium suboxides and oxygen at the surface that increased after Pt addition. This phenomenon occurred due to the reduced oxidation states of Zr and Fe. For higher oxidation states, the energy is lower, which could be associated with the correlation between the oxidation state and the binding energy.^40,41^ High binding energy band can also suggest the absorption or incorporation of OH^–^ species.^42−46^

Figure S3 shows the oxidation states of Zr species on FeZ and PFeZ. The Zr^4+^ species are related to ZrO_2_.^37,38,47^ For the FeZ sample, the curve fitting results at 181.7 eV (3d_5/2_) and 184.4 eV (3d_3/2_), which are related to the spin–orbit splitting of the Zr 3d band, relative to Zr^4+^. The shoulder at the lower energy side of the Zr 3d spectra can be related either to a minor contribution from reduced Zr species or changing of the coordination number of zirconium due to the interactions with other elements. The binding energies of the different species at the surface are presented in Table S3 of the Supporting Information. The spin–orbit splitting between Zr 3d_5/2_ and Zr 3d_3/2_ was equal to 2.5 eV, very close to the reported values in the literature, equal to 2.39^4^ and 2.4 eV,^14^ and ratio of 2:3, according with spin–orbit multiplicity.

For the mixed oxide (FeZ), there are three satisfactory fitting peaks at 178.8, 180.4, and 181.9 eV, with full width at half-maximum (fwhm) values of 2.07, 1.58, and 1.82 eV and calculated compositions of 30, 27, and 43%, respectively. Finally, the energy separations between the oxidation states were equal to 1.5 and 1.6 eV. These peaks correspond, probably, to three possible types of coordination of Zr with oxygen, not a specific oxidation state.^42^ On the other hand, the catalyst (PFeZ) shows peaks at 180.3, 181.46, and 182.42 eV, with FWHM of 1.89, 1.31, and 2.26 eV and compositions of 32.5, 19.3, and 48.2%, respectively. The energy intervals between oxidation states were equal to 1.2 and 1.3 eV.

The chemical shifts for oxides presented in the present work are similar to a previous study^48^ that found a chemical shift of ZrO_2_ around 4.6 eV of the metal oxidation state. The shifts observed at 1.3 and 3.3 eV were described by Kumar et al.^48^ and are related to suboxide I and II. Other authors reported the presence of three suboxides (Zr^+^, Zr^2+^, and Zr^3+^),^14^ corroborating with the results presented in the present work, as shown in Figure 8A,8B and Table S3 of the Supporting Information.

The XPS spectra in Figure S3 of the Supporting Information highlight the presence of Fe^3+^ and Fe^2+^. The peaks of Fe 2p_1/2_ and Fe 2p_3/2_ and satellites are presented in Table S4 of the Supporting Information for the FeZ support and the PFeZ catalyst. The Fe 2p_3/2_ has a satellite peak at about 8 eV. The peaks of Fe 2p3/2 and Fe 2p1/2 are located at 710.2 and 723.6 eV, respectively, and for the PFeZ catalyst at 710.5 and 723.9 eV. The spin–orbit splitting was equal to 13.5 eV, and the satellite peak was equal to 7.4 eV. In the present work, the fwhm of sample FeZ was larger than the fwhm of sample PFeZ because PFeZ suffered a reduction to Fe^2+^, as also described in a previous work.^40^

Figure 8B exhibits the XPS spectrum of Pt of the PFeZ catalyst, indicating bands of Pt 4f_7/2_ at 76 eV and Pt 4f_5/2_ at 73.5 eV, which can be assigned to the presence of peaks of Pt–O and PtO_2_, respectively, and a shoulder located at 70.4 eV, indicating the presence of Pt^0^, which are close to the values reported previously in the literature.^49,50^

### 3.9 DRIFTS Analyses

**Figure 9:**
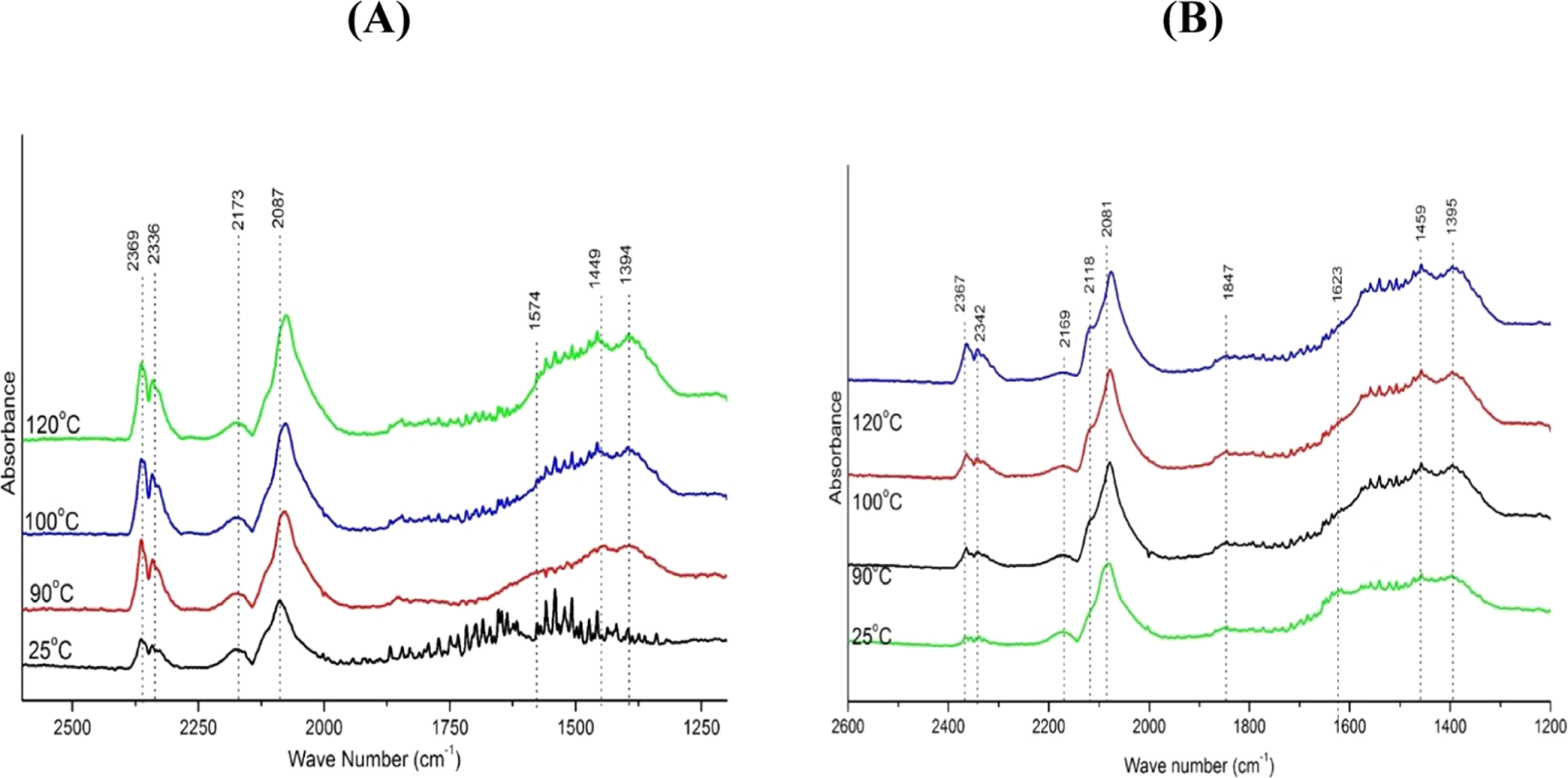
(A) CO DRIFTS spectra collected under gas flow with a 1:99 vol % CO/He feed for the PFeZ sample; (B) CO DRIFTS spectra collected under gas flow with a 1:1:98 vol % CO/O_2_/He feed for the PFeZ sample.

DRIFTS analyses were carried out to determine the intermediate species at the solid surface under similar conditions as those used in the catalytic tests. Experiments were performed with He-diluted feeds of CO, with mixtures of CO and O_2_ and mixtures of CO, O_2_, and H_2_. Results for the FeZ support treated with 1:99 vol % CO/He mixtures in a closed chamber showed hydroxyl groups (3664–3670 and 3745 cm^–1^).^51^ Carboxyl groups were observed between 1560 and 1630 cm^–1^ (*v*_as_COO^–^) and 1350 and 1420 cm^–1^ (*v*_s_COO^–^), while nonmonodentate carbonates could be seen between 1450 and 1420 cm^–1^ (*v*_as_CO_3_^2–^) and 1090 and 1020 cm^–1^ (*v*_s_CO_3_^2–^) and monodentate carbonates could be detected between 1530 and 1470 cm^–1^ (*v*_as_COO), 1300 and 1370 cm^–1^ (*v*_s_COO^–^), and 1080 and 1040 cm^–1^ (*v*C = O),^51^ as shown in Figure 9A.

DRIFTS results for the mixed oxide FeZ suggest OH^–^ oxidation by CO, leading to the formation of highly reactive compounds that can be easily oxidized by lattice O^–^, producing CO_2_ and H_2_O,^52^ in accordance with the TPD analyses. As the temperature increases, the absorption of carboxyl species also increases and consequently the formation of H_2_O.

The spectra of catalyst PFeZ are displayed in Figure 9B. The CO band indicates the adsorption on metallic sites Pt^0^ (2089 cm^–1^) and on ionic sites Pt^2+^ (2174 cm^–1^),^50,53^ suggesting adsorption double sites.^51^ The mixed oxide support (FZ) indicates the oxidation of OH^–^ with CO, leading to the formation of highly reactive compounds easily oxidized by the lattice O^2–^ and producing CO_2_ and H_2_O.^54^ One must note that the absorption band intensity of the carboxyl group observed at 1557 cm^–1^ increased with the temperature, but that the signals of the hydroxyl groups disappeared at higher temperatures.

DRIFTS absorption spectra for the FeZ mixed oxide with the feed mixture CO/O_2_/He 1:1:98 vol %, collected in the closed chamber, were similar to the previous results, showing one peak at 1625 cm^–1^, referring to the presence of a bidentate carbonate species (1620–1670 cm^–1^).^51,55^ The spectra of the PFeZ catalyst displayed a double band over Pt^0^ at 2120 and 2077 cm^–1^, as observed in Figure 9B, with significant changes when temperature increased. In contrast, the ionic M^+2^ band decreased slightly with temperature due to the oxidation of the metallic sites. The band located at 2352 cm^–1^ is related to CO_2_ in the gas phase and increased with the temperature. Also, the band located at 2120 cm^–1^ can be assigned to CO^12,52,56^

**Figure 10:**
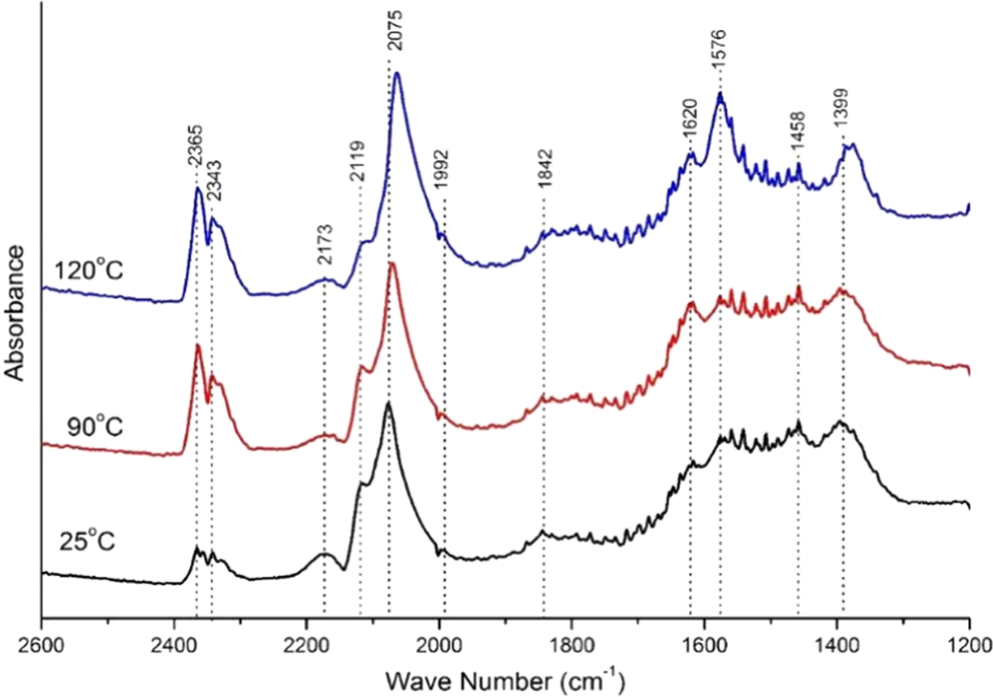
CO DRIFTS spectra collected under gas flow with CO/O_2_/H_2_/He 1:1:60:38 vol % feed for the PFeZ sample.

Figure 10 displays the DRIFTS spectra of catalyst (PFeZ) under a gas flow regime and shows monodentate carboxylates (1087 cm^–1^), nonmonodentate carboxylates (1392 cm^–1^), and noncoordinate carbonates (1459 cm^–1^). The band absorption also increased with temperature, indicating water and hydrogen formation, corroborating the observed CO-TPD results. The CO adsorption on metallic sites Pt^0^ (2089 cm^–1^) and ionic sites Pt^2+^ (2174 cm^–1^)^51^ confirm the existence of metallic Pt^0^ and Fe^2+^ ionic sites, suggesting the adsorption on double sites. Moreover, the band at 2369 cm^–1^ evidences the presence of CO_2_ and indicates the CO reaction with the lattice O^2–^.^57^ CO_2_ can also be formed by oxidation of OH^–^ with CO, resulting in CO_2_ and H_2_O.^12^ Likewise, water observed in the closed chamber condition confirms the hydroxyl oxidation hypothesis, as displayed in Figure S4(B) of the Supporting Information.

The presence of hydrogen in the feed stream affects the absorption of the PFeZ catalyst, as shown in Figure 10. In fact, CO is adsorbed as indicated by the bridged band at 1816 cm^–1^,^51^ also observed previously for Pt–Fe/alumina.^32^ One must notice the CO adsorption on metallic Pt^0^ at 2084 cm^–1^, the CO_2_ adsorption at 2345 cm^–1^, and the CO on the bridged band at 1820 cm^–1^. The CO gas adsorption band can be observed at 2119 cm^–1^, also seen for the 1:1:98 CO/O_2_/He mixture feed. Carboxylate CO_2_ species can be detected at 1560–1630 cm^–1^ (*v*COO−) and 1350–1420 cm^–1^ (*v*_s_COO−), while noncoordinate carbonates at 1458 cm^–1^ could also be observed at 120 °C.

## 4 Post-Reaction Analyses

### 4.1 Raman Spectroscopy

**Figure 11:**
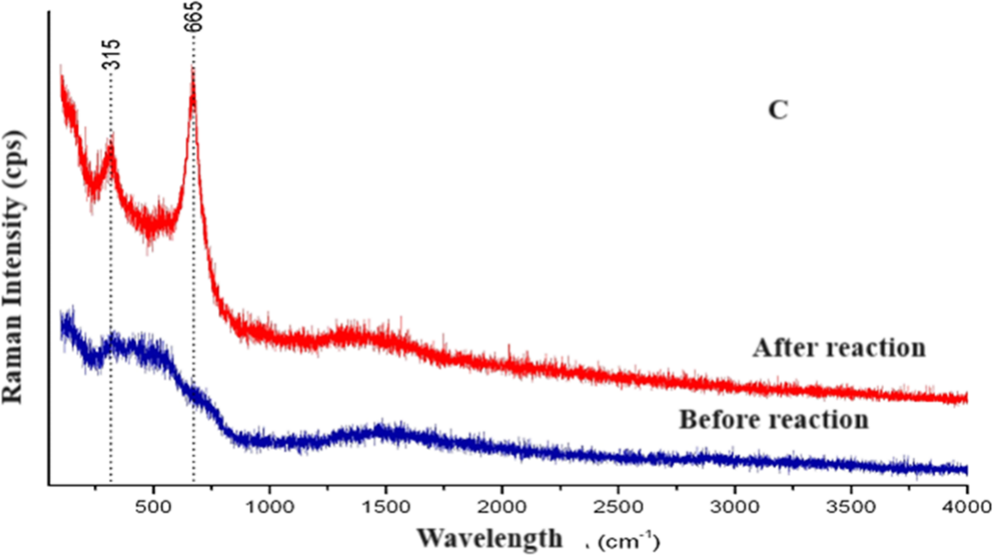
Raman spectroscopy for the sample PFeZ.

Raman spectra were recorded on a combined atomic force microscopy (AFM)-Raman System with a 532 nm excitation laser. The results are presented in Figure 11, displaying new bands after the reaction at 110 °C.

Indeed, the sample collected before the reaction did not present well-defined bands; however, after the reaction, two bands appeared at 315 and 665 cm^–1^.

Zirconia can present three crystalline forms: tetragonal, monoclinic, and cubic. The tetragonal form can be identified by bands at 270, 315, 456, 602, and 645 cm^–1^, while the bands referring to the monoclinic form can be identified at 192, 335, 347, 382, 476, 616, and 638 cm^–1^. Therefore, the band observed at 315 cm^–1^ probably represents tetragonal ZrO_2_. However, magnetite absorbs in the region of 311, 540, and 665 cm^–1^, generating a conflict of interpretation, as the bands of tetragonal zirconia and magnetite are very close. The band placed at 665 cm^–1^ probably refers to magnetite, which is expected to be located at 668 cm^–1^.^58^ In fact, the first one represents tetragonal ZrO_2_ and the latter one is related to magnetite, indicating segregation phases after the reaction. The D and G bands at around 1350 and 1580 cm^–1^ were not observed since the bands of carbon graphite (G) and defects (D) were not observed. Segregation was observed on the mixed oxide after the reaction. However, Figure 6 shows that the deactivation was very low up to 22 h of reaction, even in the presence of water in the feed stream.

### 4.2 Acid–Basic Sites

Figure S8 of the Supporting Information displays the TPD of CO_2_ of the support and catalyst for determining the basicity of the materials. The peaks of maximum of the mixed oxide and of the PFeZ catalyst were located at 130 and 103 °C, respectively, presented in Table S4 of the Supporting Information with the corresponding amounts of CO_2_, equal to 179 and 31 μmol CO_2_/g_cat_. These results indicate that the mixed oxide FZ presented higher basicity when compared to the PFeZ catalyst, probably due to blocking of the basic surface sites of the mixed oxide by PtO particles, markedly observed on the PFZ catalyst.

**Figure 12:**
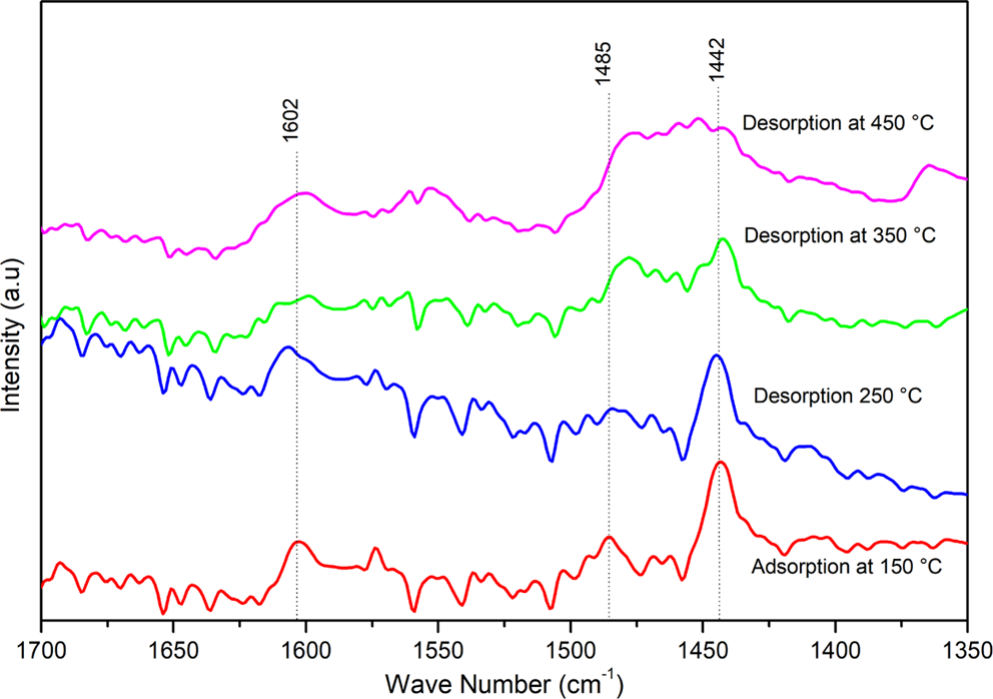
Pyridine infrared spectra of desorption at different temperatures on the PFeZ catalyst.

The Brönsted and Lewis sites acid sites were characterized by IR pyridine desorption performed at different temperatures, according to Barzetti et al.^59^Figure 12 displays the bands at different temperatures for the PFeZ catalyst, showing the presence of Lewis sites on bands located at 1442 and 1485 cm^–1^,^60^ and Brönsted bands around 1062 cm^–1^. The Lewis sites are very strong but decrease with the temperature increase. One must note that the band located at 1485 cm^–1^ increased after the addition of Pt, favoring the formation of acidic sites.

Compared to the mixed oxides, the supports became less basic after the addition of platinum, as shown in Table S4. Water was formed and adsorbed at 1602 cm^–1^.^60^

### 4.3 TPO Analyses

**Figure 13:**
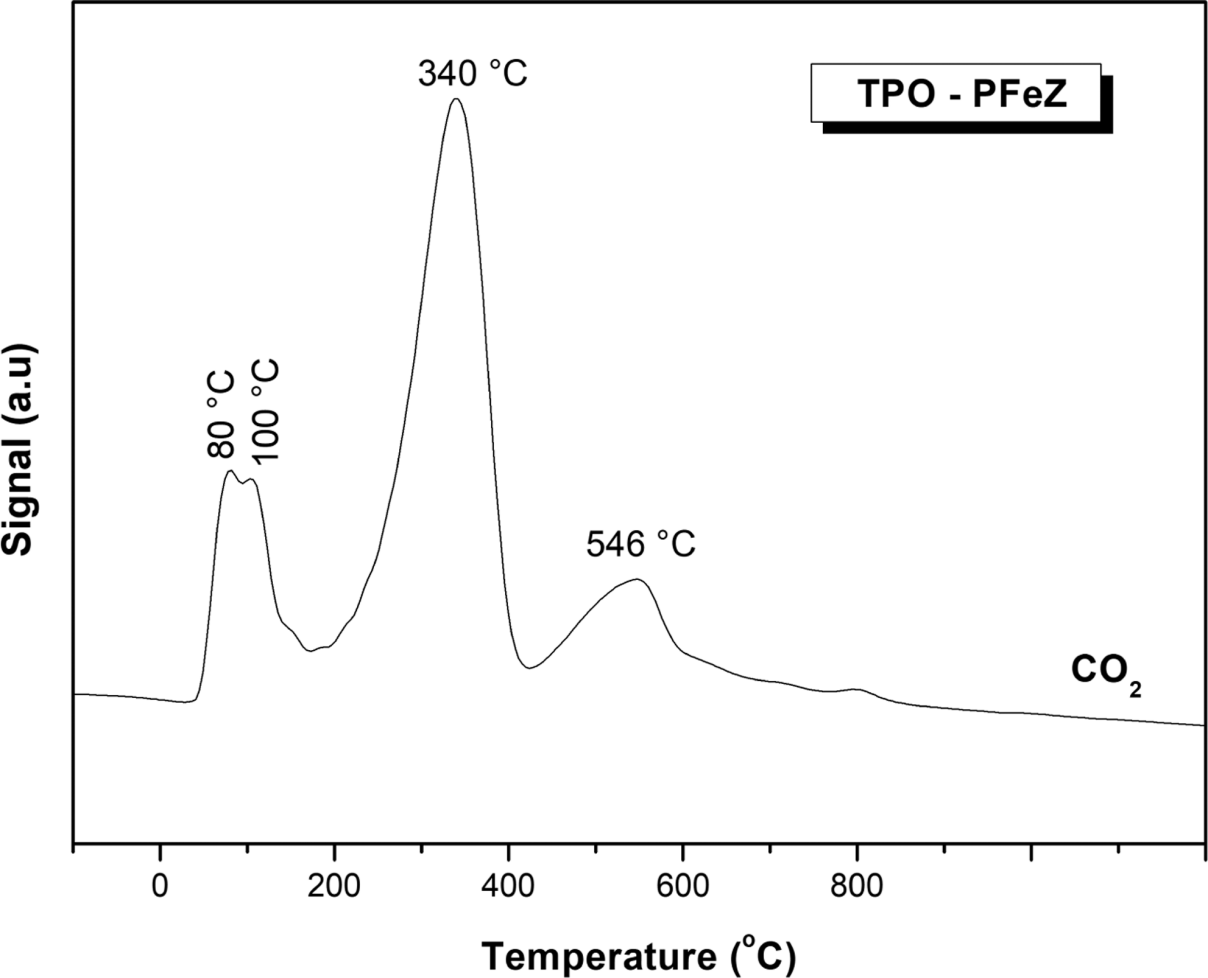
TPO profile of the PFeZ catalyst after the TP reaction with water.

These samples were also characterized through temperature-programmed oxidation after reaction in the presence of water. Results are shown in Figure 13 and Table S6 of the Supporting Information, displaying CO_2_ released after testing with water.

Figure 13 shows the CO_2_ released from the PFeZ catalyst, while Figure S7 of the Supporting Information shows the CO_2_ released from the mixed oxide (FZ) due to the coke deposition over the catalyst during the reaction, Table S6 of the Supporting Information displays the corresponding amounts of CO_2_ produced in these trials. The peaks appeared at 80, 100, 340, and 546 °C. The maximum peak for the Pt catalyst was 345 °C, when 20.9% of the CO_2_ was released, which was approximately the same amount observed with the pure mixed oxide. These results indicate low deactivation due to carbon formation and can be assigned to the carbonates sited on the mixed oxide and decomposed after TPO. The small peak located around 800 °C indicates low coke formation. These results indicate that the low deactivation due to carbon formation can be assigned to the carbonates sited on the mixed oxide and decomposed after TPO. The small peak located around 800 °C indicates low coke formation.

### 4.4 CO_2_ Temperature-Programmed Desorption

In fact, the Boudouard reaction can occur with the formation of carbon at the surface or due to the diffusion of CO into the pores of the mixed oxide. Therefore, TPO experiments were performed after the CO-TPD experiments, and the results are presented in Table 3.

The FZ sample displayed two maximum peaks at 291 and 492 °C, and the catalyst PFZ displayed three maximum peaks at lower temperatures, between 110 and 500 °C. The CO_2_ released from the FZ support was very high, about 21 μmol CO_2_/g_cat_, when compared to the PFeZ sample, about 1.78 μmol CO_2_/g_cat_ at lower temperature and 1.39 μmol CO_2_/g_cat_ at higher temperature. Thus, this indicates that carbon formation is preferentially located on the mixed oxide support. In the presence of Pt, it seems that much smaller amounts of carbon were deposited at the surface. This suggests that the dispersion of Pt inhibits the formation of carbon at the surface, which can be assigned to the blocking of the acid sites at the surface. Besides, by comparing TPO and CO-TPD results, it is possible to observe that the desorbed amounts of CO_2_ during TPD were much higher, indicating that 24% of CO_2_ desorbed in the TPD experiments corresponded to carbon deposition. On the PFZ catalyst, the release of CO_2_ was much smaller, corresponding to carbon formation around 13.3%.

### 4.5 Proposed Mechanism

**Figure 14:**
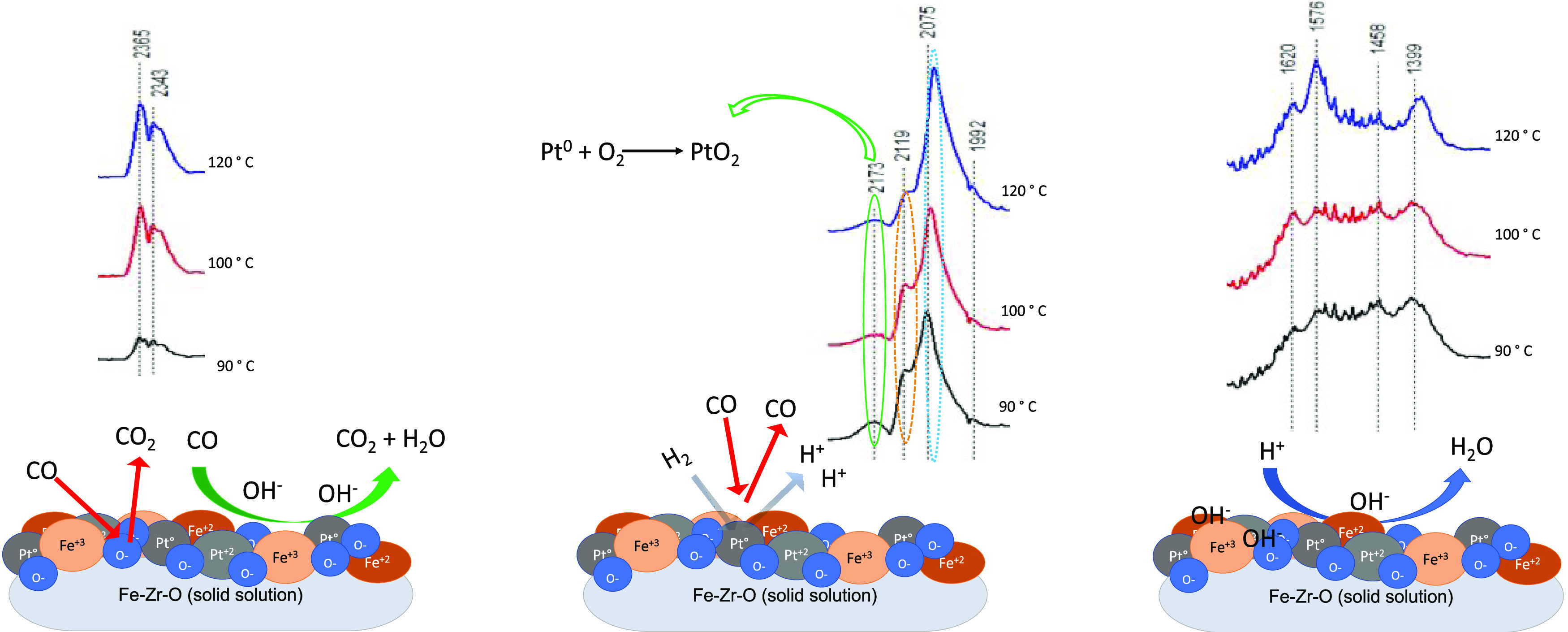
Surface reaction pathway based on XPS, TPD, and DRIFTS results.

A reaction mechanism is now proposed for the catalyst based on mixed oxide and with the addition of platinum. In fact, XPS results evidenced the presence of iron ions and the metallic form in the system. The presence of Fe and Zr ions at the surface promoted the formation of intermediates, as confirmed by the DRIFTS results. Indeed, DRIFTS results evidenced the formation of carboxylate and carbonate species, which may be formed on Fe^2+^ and Zr^+^ ions and due to the electron transfer from the molecules and interface ions, as suggested by Wu et al.^61^ Initially, oxygen adsorption occurs on Fe^2+^ ions. Then, XPS data suggest that Pt ions promote the electron transfer. The DRIFTS result indicates that oxygen adsorption takes place on Zr^2+^ at the surface, resulting in the formation of carboxylate and carbonate species at the surface, which can be oxidized with oxygen from the lattice, as shown in Figures 13 and 14. Also, the presence of OH^–^ suggests the formation of carboxyl or acetate species, according to eqs 13 and 14
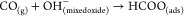
13
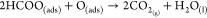
14

Afterward, the oxygen provided by the reaction leads to H_2_O formation due to the presence of hydroxyl groups on the catalyst and through oxidation of H_2_. As the temperature increases, H_2_ dissociates due to the occupation of the active sites where they compete with CO. In tests performed with CO_2_, displacement of about 10 °C of the maximum CO conversion temperature and higher selectivity were observed, suggesting that CO can be oxidized at lower temperatures due to the oxygen present in the crystalline structure. One can conclude that CO_2_ competes with O_2_ on metallic and ionic sites of the mixed oxide, thus reducing O_2_ adsorption, as shown in Figure 14.

## 5 Conclusions

Mixed oxides are active for the removal of CO contaminant from hydrogen-rich fuel cell streams due to the reduction of intermediate species of the metals, causing the CO and O_2_ adsorption onto strong and deeply reducible basic sites, as presented in CO_2_-TPD experiments. Besides, the catalytic tests and CO-TPD experiments indicate that the CO oxidation is favored by oxygen from the lattice, leading to CO_2_ formation and vacancies of the reduced species.

Platinum can interact strongly with mixed oxides, modifying the structure and surface composition of the catalyst, reducing the oxide species previously present in the sample, as determined by XPS analyses and changing the reaction mechanisms. The results indicate that the addition of Pt enhances the catalyst activity, which can be assigned to the reduction of Pt oxide, due to the presence of H_2_. Nonetheless, the decrease of CO conversion at higher temperatures favored H_2_ oxidation and CO/CO_2_ on active competitive sites.

Catalytic tests showed that the addition of platinum to oxides can reduce the maximum CO conversion temperature by more than 150 °C. In addition, CO_2_-TPD experiments suggest a drastic reduction of carbon deposition or deactivation by the blocking of PtO particles at the surface of the mixed oxides, causing a decrease of the basicity.

DRIFTS analyses showed that Pt sites preferably cause CO and H_2_ dissociation for the reaction with dissociated or molecular oxygen. Also, these sites favor the adsorption of CO at high temperatures, as determined through the increase of the band placed at 2350 cm^–1^, leading to higher rates of desorption of CO_2_ gas when the temperature increases. Moreover, the adsorption bands of CO increase with temperature over platinum while the occurrence of CO–M^+2^ bands suggests the adsorption on Fe^2+^ species. DRIFTS results evidenced the formation of carboxylate and carbonate species, which may be formed on Fe^2+^ and Zr^+^ ions and due to the electron transfer from the molecules and interface ions.

The presence of carboxyl and hydroxyl groups present at the FeZ catalyst surface and observed by DRIFTS and TPD analyses suggests the OH^–^ oxidation by CO, leading to formation of highly reactive compounds that are easily oxidized by lattice O_2_, producing CO_2_ and H_2_O. With increasing temperature, also absorbed carboxyl’s species were observed, according to the tests and TPD results, with the formation of H_2_O.

As a whole, it is possible to analyze the reaction mechanism that leads to CO consumption using a catalyst based on mixed oxides with the addition of platinum. It can be concluded that CO_2_ competes with O_2_ on metallic sites, thus reducing O_2_ adsorption.

## Supporting Information Available

Data for surface areas; XPS; DRIFTs; and activity tests of samples before and after reaction (PDF)
