# Supplementary material for: Effects of Metal Promoters (M = Fe, Co, and Cu) in Pt/MxZryOz Catalysts and Influence of CO2 and H2O on the CO Oxidation Activity (PROX): Analysis of Surface Properties After Reaction
Source: ACS Omega. 2024 Jun 7;9(24):25715–29. doi: 10.1021/acsomega.3c09039 (PMC11190919; doi:10.1021/acsomega.3c09039)
Supplement: Supplementary file 1 — ao3c09039_si_001.pdf [file ao3c09039_si_001.pdf]

SUPPLEMENTAR INFORMATION

Effects of Metal Promoters (M = Fe, Co and Cu) in Pt/$M_xZr_yO_z$ Catalysts and Influence of CO_2 and H_2O on the CO Oxidation Activity (PROX): Analysis of Surface Properties After Reaction

*Carolina C. Gaioto[1], José Carlos Pinto[1], Martin Schmal[1,2]**

[1]Programa de Engenharia Química / COPPE, Universidade Federal do Rio de Janeiro, Cidade Universitária, CP: 68502, Rio de Janeiro - 21941-972 RJ, Brazil
[2]Departamento de Engenharia Química, Universidade de São Paulo, Cidade Universitária, São Paulo – SP, Brazil

Figures SI

Figure S1 - Isotherms of FZ and CuZ samples and of the catalysts.

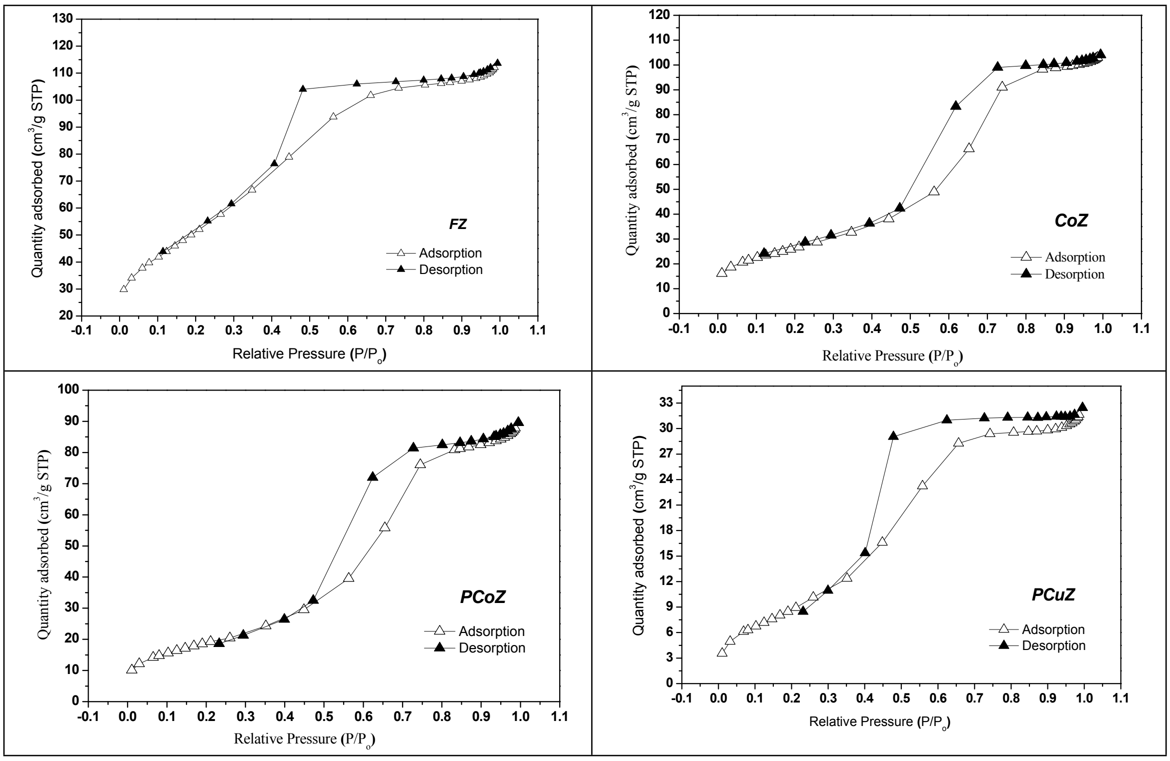

Figure S2 - CO_2 desorption profile of support and catalyst after CO adsorption. (A) – PFeZ; (B) – FZ.

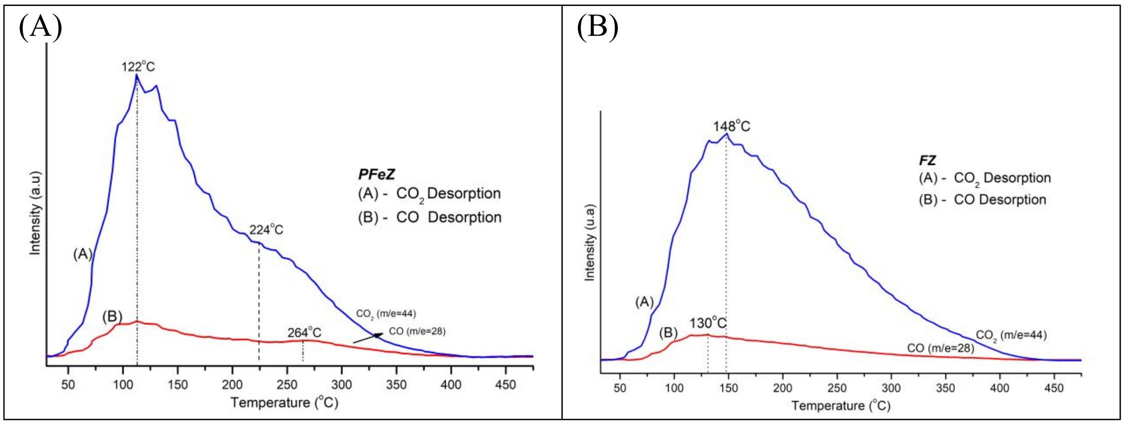

Figure S3 – (A) Iron Fe 2P3/2 and Fe 2P1/2 XPS spectra of samples FeZ and PFeZ; (B) Zirconium Zr 3d$_{5/2}$ and Zr 3d$_{3/2}$ XPS spectra of samples FeZ and PFeZ.

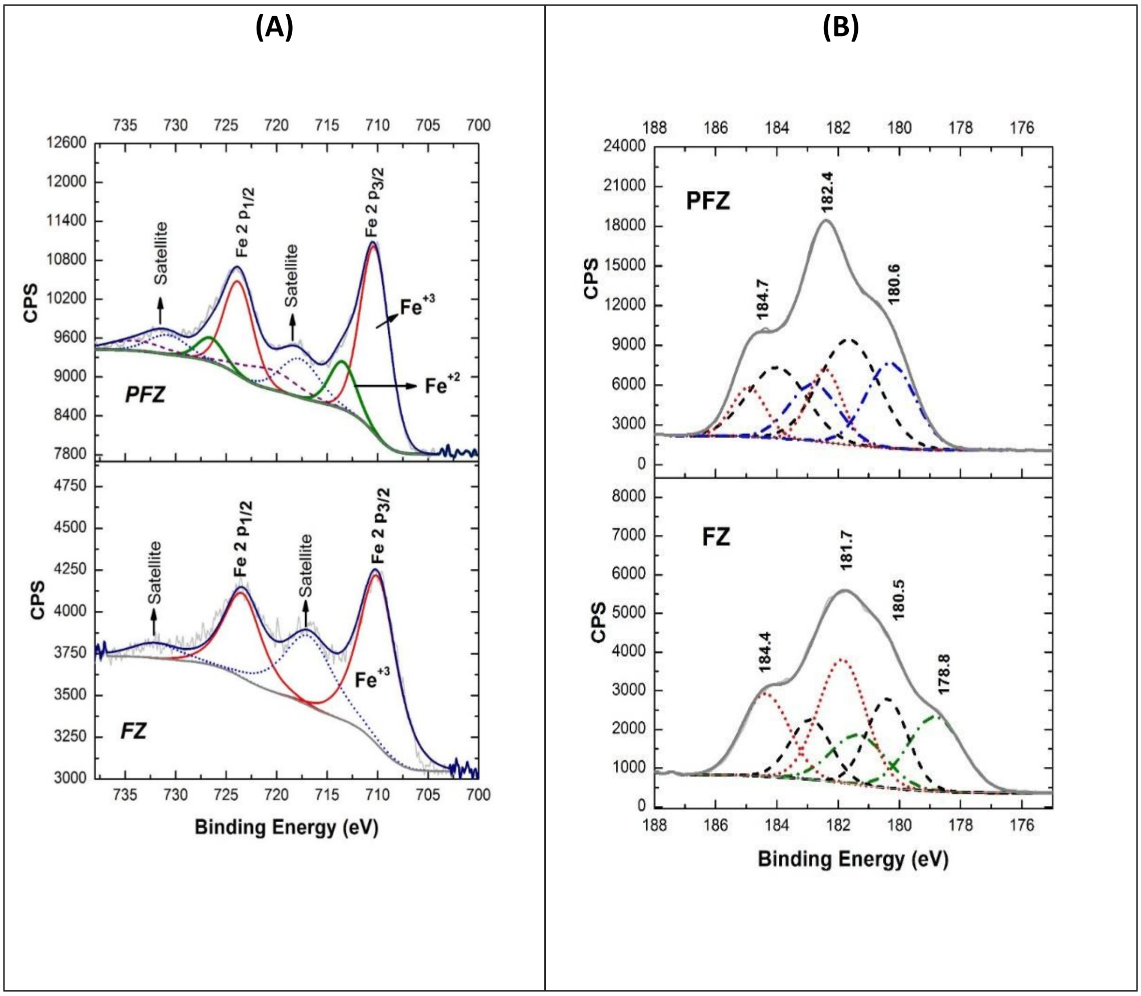

Figure S4 – (A) **-** CO DRIFTS spectra collected in closed chamber with CO/He 1:99 mol% feed for the FeZ; (B) - CO DRIFTS spectra collected in closed chamber with CO/He 1:99 mol% feed for the PFeZ sample.

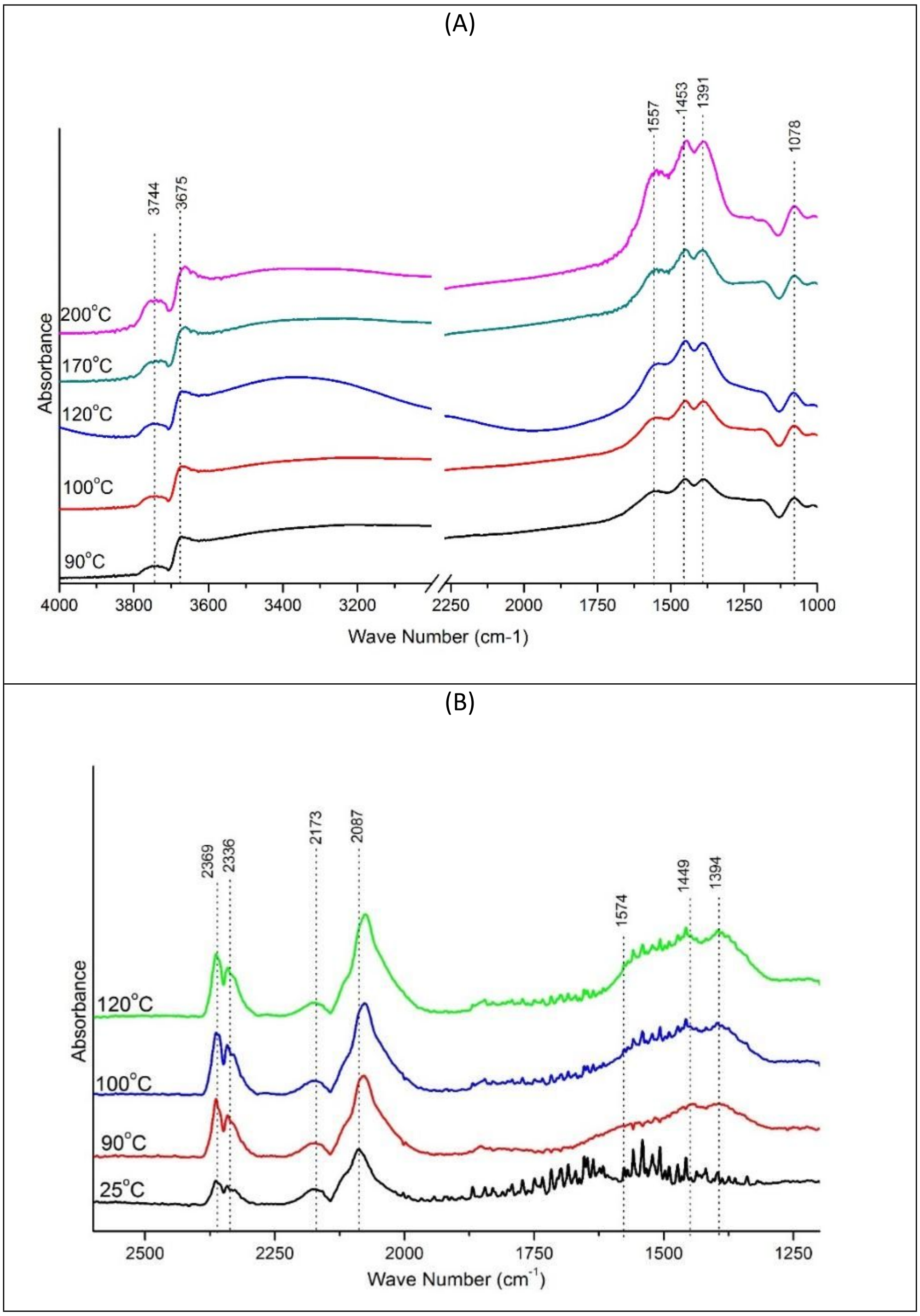

Figure S5 - Selectivity of CO_2 of mixed oxides and catalysts for the Prox reaction.

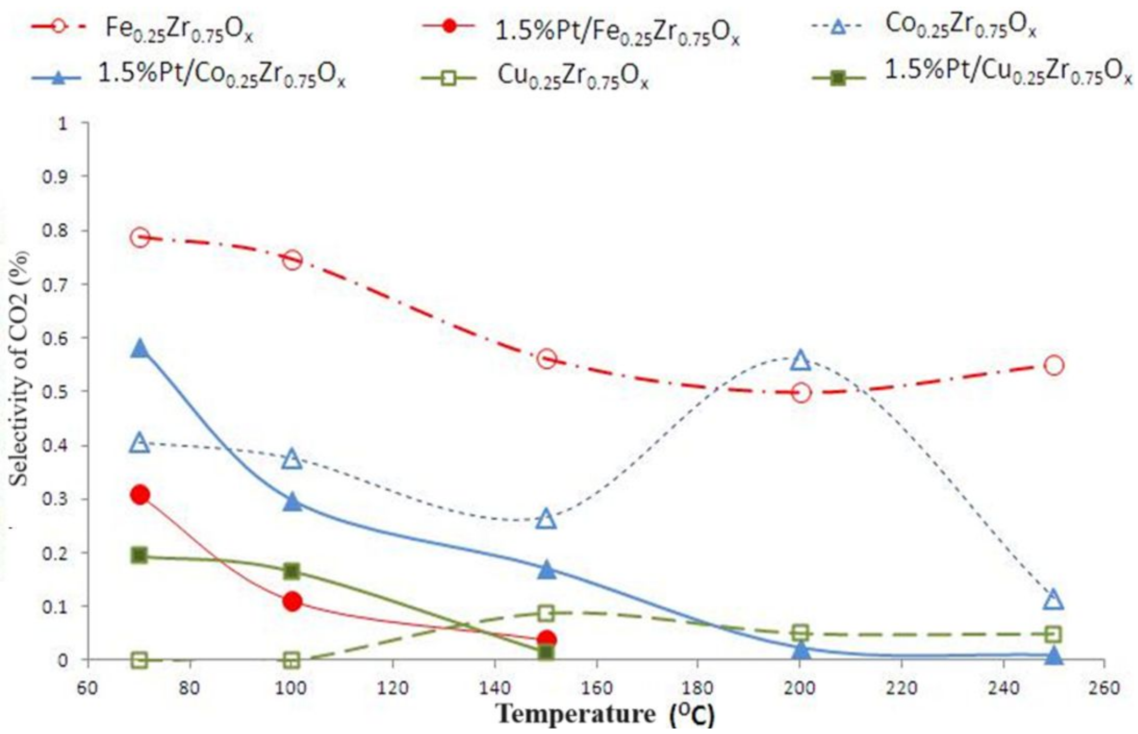

Figure S6 -TPSR profiles in the presence or not of CO_2 and H_2O.

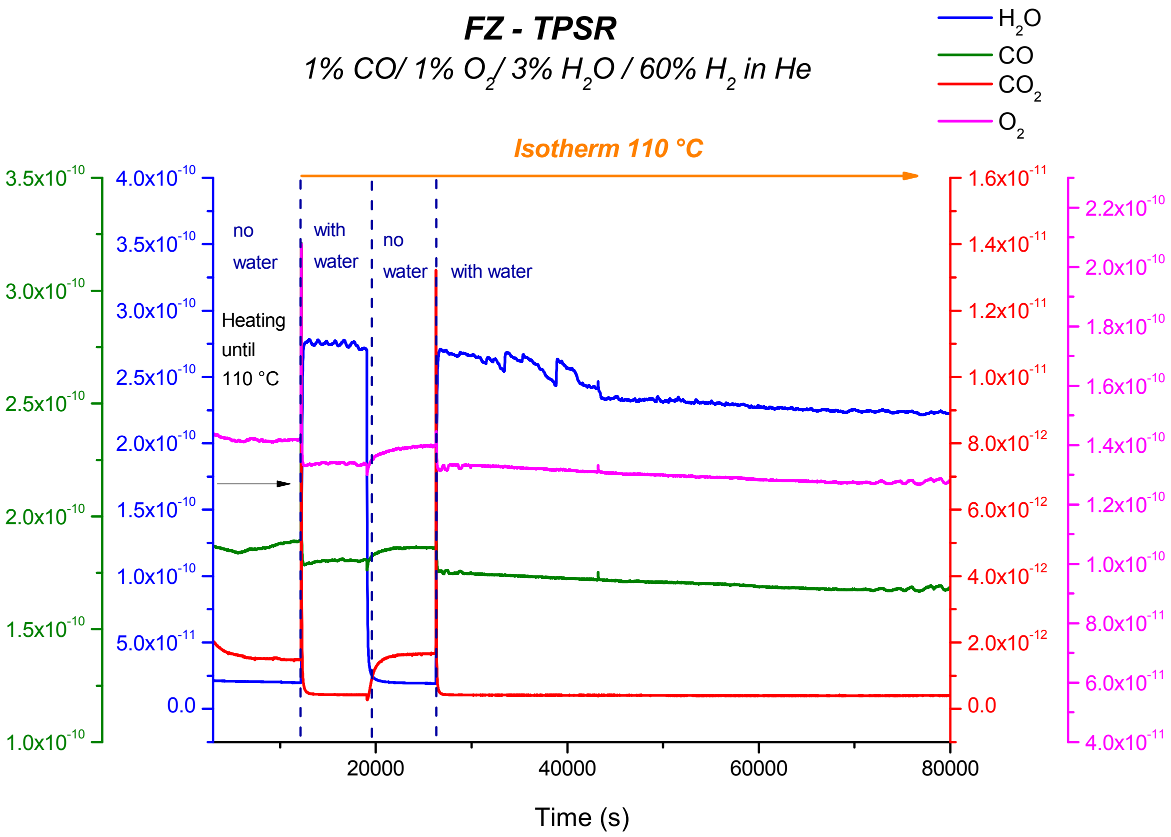

Figure S7 - TPO profile of FZ after TPRe with water.

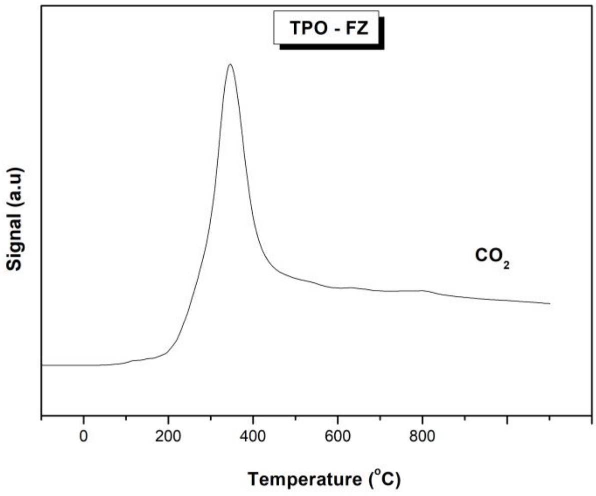

Figure S8 - Desorption profiles of CO_2 for the mixed oxide (FZ) and the catalyst (PFeZ).

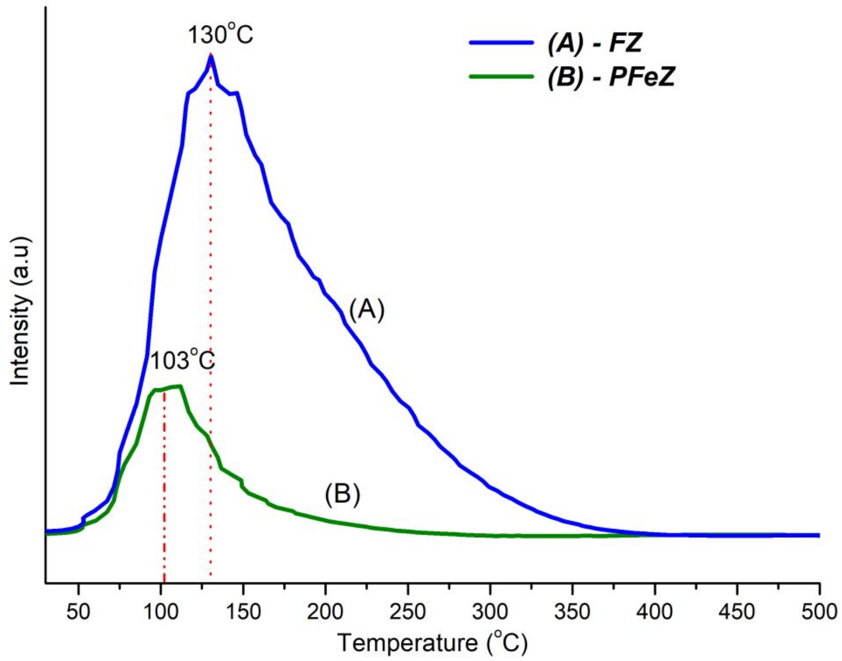

Tables

Table S1 - Nomenclature and chemical composition of the catalyst and support.

System	Nominal content (wt.%)			Real content (wt.%)			
	Pt	ZrO_2	Fe_2O_3	Pt	ZrO_2	Fe_2O_3	Cl
$FeZrO_2$	-	-	-		**76**	**20**	**-**
Pt-$FeZrO_2$	1.5	2.3	z	1.7	73	19	

Table S2 - TPR of hydrogen consumption results.

	PFeZ	FeZ
**µmol/g_{cat} Total**	200.93	191.33
**Pt/Fe or Fe**	62. 9 (~31%)	63.4 (~33%)
**Zr**	138.03 (~69%)	128 (~67%)

Table S3 - Superficial composition from EDS analyses.

Element	***FeZ***	***PFeZ***	***FeZ***	***PFeZ***
	**Weight%**		**Atomic%**	
O	27.8	38.8	66.4	76.5
Fe	12.8	12.0	8.7	6.8
Zr	59.4	47.9	24.9	16.5
Pt		1.3		0.2
Total	100.0	100.0	100.0	100.0

Table S4 - Quantitative desorption analyses of CO_2 of the mixed oxide support and the catalyst.

Sample	Temperature (°C)	(µ mol CO2 /g $_{cat}$)
FZ	130	179
PFeZ	103	31

Table S5 – XPS spectral values of Platinum.

	Pt $4f_{7/2}$ (eV)	Pt $4f_{5/2}$ (eV)	FWHM (eV)	Ref.
Pt^0	70.4	73.8	2.6	This work
PtO	73.2	76.6	3.1	This work
PtO_2	75.6	79.0	1.9	This work
Pt^0	70.9	74.2		[51]
PtO	~73.0			[50]
PtO_2	~74.5-75.0			[50]
Pt^0*	71.8			[50]
PtO*	73.2			[50]
PtO_2*	74.6			[50]

* Pt/Sibunit

Table S6 – TPO of FeZ and PFeZ after reaction.

**Samples**	**CO_2 released (µmol/g)**			
	**Peak 80°C**	**Peak 100°C**	**Peak 350°C**	**Peak 548°C**
FeZ	-	-	108.3	-
PFeZr	6.0	30.8	105.5	33.6

S7 - Calculation of the surface metallic area and dispersion

To calculate the metallic area, the following expression was used:

$$S'_m = \frac{N_s}{[L]}$$

where **[L]** is the maximum site density, or the number of surface metal atoms per m^2. For platinum, the value of **[L]** is equal to 0.96×10^{19} atoms/m^2. $\mathbf{N_s}$ is the number of surface sites; that is, the number of chemically adsorbed molecules. Considering the metal content, we have:

$$S_m = \frac{N_s}{y.[L]}$$

The dispersion was calculated in accordance with the following equation:

$$D(\%) = \frac{N_s}{N_t}.100$$

where D is the dispersion and Nt is the total number of atoms, given by the following equation:

$$N_t = \frac{y.m_{cat}}{M}.N_A$$

where NA is the Avogadro number, y is the fraction of the supported metal, mcat is the catalyst mass and M is the atomic mass of the metal considered.

Turnover Frequency (TOF)

The turnover frequency (TOF) was formulated as the intrinsic activity of the site, expressed as: TOF = Rate of component j formation / active site.

$$TOF\ (s^{-1}) = Fj.N_A/S_i$$

where Fj represents the molar flow rate (mol/s), N_A denotes Avogadro's number (6.023x1023 molecules), and Si represents an active site = [L].[S], with [L] representing atom density (atoms/m^2) and [S] representing surface area (m^2).

Figure Captions in SI

Figure S1 - Isotherms of FZ and CuZ samples and of the catalysts.

Figure S2 - CO_2 desorption profile of support and catalyst after CO adsorption.

Figure S3 - Iron Fe 2P3/2 and Fe 2P1/2 XPS spectra of samples FeZ and PFeZ; B: Zirconium Zr 3d5/2 and Zr 3d3/2 XPS spectra of samples FeZ and PFeZ.

Figure S4 - CO DRIFTS spectra collected in closed chamber with CO/He 1:99 mol% feed for the FeZ sample– (B) CO DRIFTS spectra collected in closed chamber with CO/He 1:99 mol% feed for the PFeZ sample.

Figure S5 - Selectivity of CO_2 of mixed oxides and catalysts for the Prox reaction.

Figure S6 -TPSR profiles in the presence or not of CO_2 and H_2O.

Figure S7 – TPO profile of FZ after TPRe with water.

Figure S8 - Desorption profiles of CO_2 for the mixed oxide (FZ) and the catalyst (PFeZ).

Table Captions

Table S1 - Nomenclature and chemical composition of the catalyst and support.

Table S2 - TPR hydrogen consumption results.

Table S3 - The superficial composition from EDS analyses.

Table S4 - Quantitative desorption analyses of CO_2 of the mixed oxide support and the catalyst.

Table S5 – XPS spectral values of Platinum.

Table S6 – TPO of the FeZ and PFeZ catalyst after reaction.

S7 - Calculation of the surface metallic area and dispersion
